# Relationship Between Blood Pressure and Incident Cardiovascular Disease: Linear and Nonlinear Mendelian Randomization Analyses

**DOI:** 10.1161/HYPERTENSIONAHA.120.16534

**Published:** 2021-04-05

**Authors:** Rainer Malik, Marios K. Georgakis, Marijana Vujkovic, Scott M. Damrauer, Paul Elliott, Ville Karhunen, Alice Giontella, Cristiano Fava, Jacklyn N. Hellwege, Megan M. Shuey, Todd L. Edwards, Tormod Rogne, Bjørn O. Åsvold, Ben M. Brumpton, Stephen Burgess, Martin Dichgans, Dipender Gill

**Affiliations:** 1 Institute for Stroke and Dementia Research, University Hospital, Ludwig-Maximilians-University LMU, Munich, Germany (R.M., M.K.G., M.D.).; 2 Perelman School of Medicine, University of Pennsylvania, Philadelphia (M.V., S.M.D.).; 3 Corporal Michael Crescenz Veterans Affairs Medical Center, Philadelphia, PA (M.V., S.M.D.).; 4 Department of Epidemiology and Biostatistics, School of Public Health (P.E., V.K., D.G.), Imperial College London, United Kingdom.; 5 British Heart Foundation Centre of Research Excellence (P.E., D.G.), Imperial College London, United Kingdom.; 6 School of Public Health, Medical Research Council-Public Health England Centre for Environment (P.E.).; 7 UK Dementia Research Institute at Imperial College London, United Kingdom (P.E.).; 8 Imperial Biomedical Research Centre, Imperial College London and Imperial College NHS Healthcare Trust, United Kingdom (P.E.).; 9 Health Data Research UK London (P.E.).; 10 Department of Medicine, University of Verona, Italy (A.G., C.F.).; 11 Department of Clinical Sciences, Clinical Research Centre, Lund University, Malmö, Sweden (A.G., C.F.).; 12 Division of Genetic Medicine, Department of Medicine, Vanderbilt Genetics Institute, Vanderbilt Epidemiology Center (J.N.H., M.M.S.) Vanderbilt University Medical Center, Nashville, TN.; 13 Division of Epidemiology, Department of Medicine, Vanderbilt Genetics Institute (T.L.E.), Vanderbilt University Medical Center, Nashville, TN.; 14 Department of Circulation and Medical Imaging, Gemini Center for Sepsis Research, Norwegian University of Science and Technology, Trondheim, Norway (T.R.).; 15 Department of Chronic Disease Epidemiology, Yale University School of Public Health, New Haven, CT (T.R.).; 16 Clinic of Anaesthesia and Intensive Care (T.R.), St. Olav’s Hospital, Trondheim University Hospital, Norway.; 17 Department of Endocrinology, Clinic of Medicine (B.O.Å.), St. Olav’s Hospital, Trondheim University Hospital, Norway.; 18 Clinic of Thoracic and Occupational Medicine (B.M.B.), St. Olav’s Hospital, Trondheim University Hospital, Norway.; 19 Department of Public Health and Nursing, K.G. Jebsen Center for Genetic Epidemiology, Norwegian University of Science and Technology (B.O.Å., B.M.B.).; 20 Cardiovascular Epidemiology Unit, University of Cambridge, United Kingdom (S.B.).; 21 Medical Research Council Biostatistics Unit, University of Cambridge, United Kingdom (S.B.).; 22 Munich Cluster for Systems Neurology, Germany (M.D.).; 23 German Centre for Neurodegenerative Diseases, Munich, Germany (M.D.).; 24 Clinical Pharmacology and Therapeutics Section, Institute of Medical and Biomedical Education and Institute for Infection and Immunity, St George’s, University of London, United Kingdom (D.G.).; 25 Clinical Pharmacology Group, Pharmacy and Medicines Directorate, St George’s University Hospitals NHS Foundation Trust, London, United Kingdom (D.G.).; 26 Novo Nordisk Research Centre Oxford, Old Road Campus, United Kingdom (D.G.).

**Keywords:** blood pressure, coronary artery disease, hypertension, primary prevention, stroke

## Abstract

Observational studies exploring whether there is a nonlinear effect of blood pressure on cardiovascular disease (CVD) risk are hindered by confounding. This limitation can be overcome by leveraging randomly allocated genetic variants in nonlinear Mendelian randomization analyses. Based on their association with blood pressure traits in a genome-wide association study of 299 024 European ancestry individuals, we selected 253 genetic variants to proxy the effect of modifying systolic and diastolic blood pressure. Considering the outcomes of incident coronary artery disease, stroke and the combined outcome of CVD, linear and nonlinear Mendelian randomization analyses were performed on 255 714 European ancestry participants without a history of CVD or antihypertensive medication use. There was no evidence favoring nonlinear relationships of genetically proxied systolic and diastolic blood pressure with the cardiovascular outcomes over linear relationships. For every 10-mm Hg increase in genetically proxied systolic blood pressure, risk of incident CVD increased by 49% (hazard ratio, 1.49 [95% CI, 1.38–1.61]), with similar estimates obtained for coronary artery disease (hazard ratio, 1.50 [95% CI, 1.38–1.63]) and stroke (hazard ratio, 1.44 [95% CI, 1.22–1.70]). Genetically proxied blood pressure had a similar relationship with CVD in men and women. These findings provide evidence to support that even for individuals who do not have elevated blood pressure, public health interventions achieving persistent blood pressure reduction will be of considerable benefit in the primary prevention of CVD.

## Abstract 2

Supplemental Digital Content is available in the text.

More than 1 billion people worldwide experience hypertension,^1^ which is estimated to account for >20% of cardiovascular disease (CVD).^2^ Meta-analyses of randomized controlled trials have shown that a 10-mm Hg reduction in systolic blood pressure (SBP) is associated with a 15% to 20% reduction in the risk of coronary artery disease (CAD) and a 25% to 30% reduction in the risk of stroke.^3^ As such, blood pressure lowering is one of the most effective strategies for reducing the burden of CVD.^4,5^

Large observational studies have previously explored the relationship between blood pressure and cardiovascular risk, potentially identifying linear associations in individuals free of CVD at baseline^6,7^ but J-shaped associations both in the general population^8^ and in patients with a history of CAD^9^ and stroke.^10^ However, it is difficult to make causal conclusions about the effects of altering blood pressure from such data because any identified associations may be susceptible to confounding from unknown or unmeasured factors. For the patients with elevated cardiovascular risk recruited to the SPRINT (Systolic Blood Pressure Intervention Trial), SBP lowering to <120 mm Hg as compared with 140 mm Hg resulted in fewer major cardiovascular events.^11^ However, no high-quality clinical trials have investigated the effect of blood pressure lowering below this level. Excessive blood pressure reduction in patients with atherosclerotic disease can reduce organ perfusion and increase CVD risk.^12^ Insight into the shape of the relationship between blood pressure and CVD risk is, therefore, critical for informing optimal prevention strategies.

In the Mendelian randomization (MR) paradigm, genetic variants can be used as proxies for studying the effect of varying blood pressure.^13^ In the same way as treatment allocation in a randomized controlled trial setting, random allocation of genetic variants means that they are unlikely to be affected by confounding from environmental factors.^14^ Recent methodological developments have allowed for MR investigation into the shape of the relationship between risk factors and outcomes.^15–17^ In this study, we use MR to investigate the shape of the relationship between genetically proxied blood pressure and incident CVD in a general population without a history of CVD or antihypertensive medication use. Our analyses aim to provide novel insight that can be used to inform public health strategies toward the primary prevention of CVD.

## Methods

All data supporting the findings of this study are available from the corresponding author upon reasonable request. The UK Biobank study was approved by the North West Multicentre Research Ethics Committee, and all participants provided informed consent. All variants used as instruments in this study and their genetic association estimates are provided in the Data Supplement. All results from the analyses performed in this work are presented in the main article or its Data Supplement. This article has been reported based on recommendations by the STROBE-MR Guidelines (Checklist in the Data Supplement).^18^ The study protocol and details were not preregistered.

### UK Biobank

The UK Biobank cohort is comprised of ≈500 000 people (94% of self-reported European ancestry) aged 40 to 69 years at baseline and recruited between 2006 and 2010 at 22 assessment centers throughout the United Kingdom. Participants were followed up until January 1, 2018, or their date of death. Along with genotyping, the resource has information on clinical measurements, assays of biological samples, and self-reported health behavior. Moreover, it is supplemented by linkage with electronic health records including hospital inpatient data, mortality data, and cancer registries.^19^

For the exposures of interest, SBP and diastolic blood pressure (DBP), data were collected using an automated reading when participants attended the assessment center for baseline measurements (UK Biobank fields 4080 for SBP and 4079 for DBP). When multiple baseline measurements were available, the mean of the measured values was used.

As our primary outcomes, we selected a combined incident cardiovascular end point of CAD and stroke (referred to hereafter as CVD), incident CAD, and incident stroke. We used hospitalization-based *International* *Classification of Diseases*, *Tenth Revision*, and Office of Population Censuses and Surveys Classification of Surgical Operations and Procedures (fourth revision) codes to identify events (Table S1 in the Data Supplement). For individuals with multiple incident events (eg, incident CAD and incident stroke), the first event recorded was used. Related individuals (kinship coefficient, >0.0884) and those with prevalent CVD (identified through hospitalization codes and self-report) were excluded from the analyses. Individuals taking antihypertensive medications at baseline (UK Biobank field 20003) were also excluded from the analyses because their observed blood pressure is not reflective of their genetically predicted blood pressure, thus introducing bias into the nonlinear MR estimates.^15,17^

### Candidate Instrumental Variables

For our primary analysis, we selected 253 uncorrelated (r^2^<0.1) single-nucleotide polymorphisms as candidate instrumental variables for SBP and DBP based on their previously published associations with blood pressure traits.^20^ Their associations with SBP and DBP were estimated in a genome-wide association study of 299 024 European ancestry individuals performed by the International Consortium of Blood Pressure study, which did not include UK Biobank participants.^20^ Using the coefficients for association with SBP and DBP (Table S2), a weighted allele score for each participant was created by multiplying the blood pressure–increasing allele dosage with the variant’s association with SBP or DBP, respectively, and summing across the 253 variants. The above genetic association estimates were taken from a study that adjusted for body mass index. As this could theoretically bias the analyses,^21^ we further performed a sensitivity analysis that selected variants from a genome-wide association study meta-analysis of 2 non-UK Biobank cohorts that did not adjust for body mass index (n=122 361; Methods in the Data Supplement). Fixed-effects meta-analysis was performed using METAL,^22^ and variants reaching genome-wide significance (*P*<5×10^−8^) were clumped to correlation r^2^<0.01 using PLINK.^23^ We extracted 22 uncorrelated variants as instrumental variables for SBP and 27 uncorrelated variants as instrumental variables for DBP in this sensitivity analysis (Tables S3 and S4).

### Statistical Analyses

All statistical analyses were performed using R (version 3.6.2). Differences in characteristics between UK Biobank population subgroups were assessed using a Student *t* test, Wilcoxon rank-sum test, Fisher exact test, or χ^2^ test as appropriate. We performed MR analyses investigating the association between genetically proxied blood pressure (either SBP or DBP) and incident CVD, CAD, and stroke risk. Analyses were performed by modeling a linear relationship between genetically proxied blood pressure and the outcomes (linear MR)^14,24^ and also using the fractional polynomial method to test for a nonlinear relationship between genetically proxied blood pressure and the outcomes (nonlinear MR).^15,17^

#### Linear MR

We used the ratio of coefficients method to perform MR analyses that assumed a linear association of genetically proxied blood pressure with the risk of incident CVD, CAD, and stroke.^25^ This represents the association of the allele score with the cardiovascular outcome (incident CVD, CAD, or stroke) divided by the association of the allele score with the blood pressure trait (either SBP or DBP).^26^ Linear regression was used to estimate the association of the allele score with blood pressure, incorporating age, sex, principal components 1 to 10 of genetic ancestry, genotyping chip, and assessment center as covariates. The proportion of blood pressure variance explained by the allele score and its F statistic were calculated to estimate instrument strength.^27^ Cox proportional hazard regression was used to estimate the association of the allele score with the outcomes, incorporating age, sex, principal components 1 to 10 of genetic ancestry, genotyping chip, and assessment center as covariates. As sensitivity analyses, we considered each variant in the allele score separately and performed MR methods that differ in their requisite assumptions regarding the inclusion of pleiotropic variants: random-effects inverse-variance weighted MR, MR-Egger, weighted median MR, and MR-PRESSO.^28^ An intercept term in MR-Egger differing from zero can be used to evidence the presence of directional pleiotropy,^29^ and MR-PRESSO is able to identify variants with outlying estimates that may in turn be excluded from analyses.^30^

#### Nonlinear MR

We applied the fractional polynomial method to investigate for evidence of a nonlinear relationship between genetically proxied blood pressure and risk of incident CVD, CAD, and stroke. This approach has been described previously in detail^15–17^ and is outlined in Methods in the Data Supplement. Briefly, we stratified the population into centiles based on residual blood pressure, defined as a participant’s blood pressure minus the genetic contribution to blood pressure from the allele score. By doing this, we aimed to compare individuals in the population who would have similar blood pressure values (values in the same centile) if they had the same genetic predisposition. Stratifying on blood pressure directly would introduce collider bias to distort estimates, as blood pressure is on the causal pathway from the genetic variants to CVD.^17,31^ For each centile, we calculated a linear MR estimate for the association of genetically proxied blood pressure with the outcome using the ratio of coefficients method, as described above.^26^ Using a flexible semiparametric framework, we then performed a meta-regression of the linear MR estimates obtained for each centile against the mean blood pressure in that centile.^16,17^ A fractional polynomial test was used to investigate whether a nonlinear model fit this meta-regression better than a linear model (further detailed in Methods in the Data Supplement). A Bonferroni correction was applied to account for multiple testing of the 2 blood pressure traits and 3 outcomes, with *P*<8×10^−3^ representing statistical significance. We further conducted a priori–specified subgroup analyses considering men and women separately to investigate potential sex-specific effects.

Individuals with elevated blood pressure are more likely to be prescribed antihypertensive medications, and, therefore, exclusion of these individuals from the main analysis could potentially distort MR estimates due to selection effects and introduction of collider bias. Inverse probability weighting was, therefore, performed in a sensitivity analysis to investigate this, as described in Methods in the Data Supplement.

## Results

A total of 255 714 participants were included in analyses, after excluding 66 011 individuals with a history of antihypertensive medication use and 6506 individuals with a history of CVD (but not on antihypertensive medications). There were 10 606 incident CVD events, including 8430 incident CAD events (68.1% *International Classification of Diseases*, *Tenth Revision*, based) and 2176 incident stroke events. The allele score explained 4.8% and 4.5% of the variance for SBP and DBP, respectively, corresponding to F statistics of 58.6 and 54.1 and low risk of substantial weak instrument bias. The distribution of CVD risk factors for individuals in the analyzed population that had a weighted allele score for SBP and DBP above and below the population median in the main and sensitivity analyses is provided in Table 1. Table S5 provides these data for individuals in the top and bottom deciles of residual blood pressure in the main analysis.

**Table 1.**
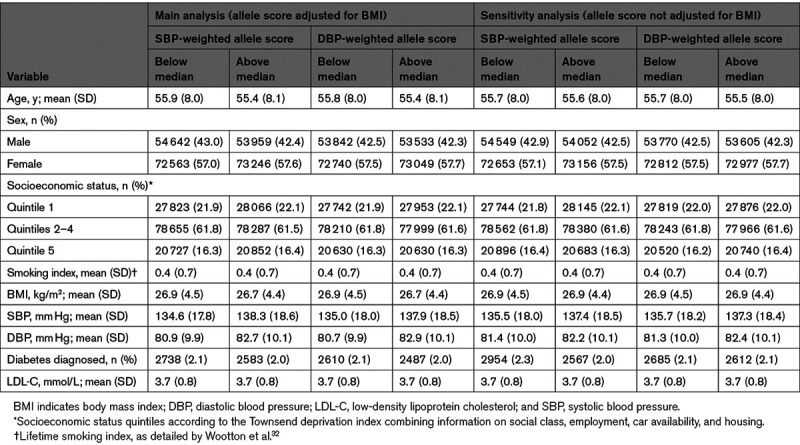
Distribution of Risk Factors for Individuals in the Analyzed Population That Had a Weighted Allele Score for SBP and DBP Above and Below the Population Median in the Main and Sensitivity Analyses

### Linear MR

Linear MR analyses identified a strong association of both genetically proxied SBP and DBP with the cardiovascular outcomes. For a 10-mm Hg increase in genetically proxied SBP, the hazard ratio (HR) of incident CVD was 1.49 ([95% CI, 1.38–1.61] *P*=7×10^−25^), incident CAD was 1.50 ([95% CI, 1.38–1.63] *P*=2×10^−21^), and incident stroke was 1.44 ([95% CI, 1.22–1.70] *P*=1×10^−5^). For a 5-mm Hg increase in genetically proxied DBP, the HR of incident CVD was 1.35 ([95% CI, 1.29–1.42] *P*=5×10^−34^), incident CAD was 1.36 ([95% CI, 1.26–1.47] *P*=1×10^−15^), and incident stroke was 1.39 ([95% CI, 1.20–1.62] *P*=2×10^−5^). The MR-Egger test did not detect significant directional pleiotropy (Table S6), and MR-PRESSO only identified 16 single-nucleotide polymorphisms as outliers in the analysis of genetically proxied SBP and CAD (Table S2). Similar MR estimates were obtained in sensitivity analyses (Table S6; Figures S1 and S2).

### Nonlinear MR

**Figure 1.**
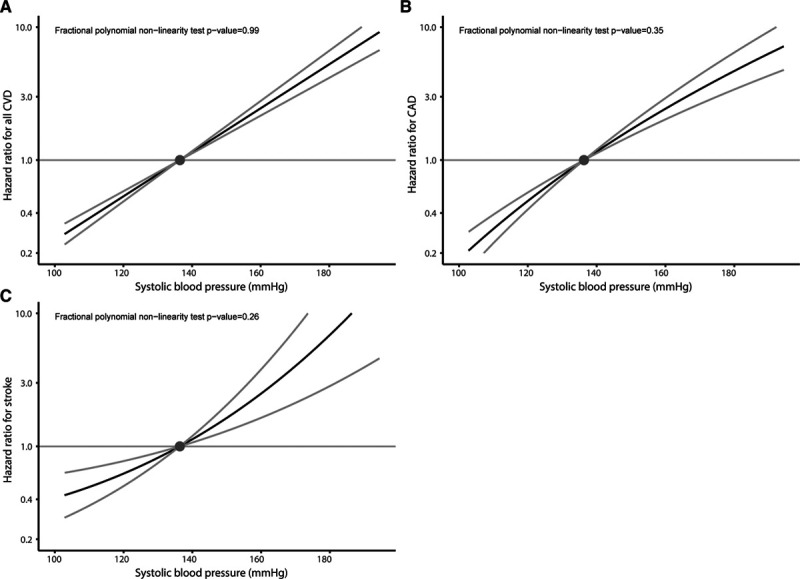
**Nonlinear Mendelian randomization considering genetically proxied systolic blood pressure (SBP) and incident cardiovascular outcomes.** Nonlinear Mendelian randomization considering genetically proxied systolic blood pressure (SBP) and incident cardiovascular outcomes: (**A**) all incident cardiovascular disease (CVD) events, (**B**) incident coronary artery disease (CAD), and (**C**) incident stroke. Displayed on the *x* axis are SBP values in mm Hg. The *y* axis shows the hazard ratio for the respective incident cardiovascular event. Reference is set to a population mean SBP value of 136.5 mm Hg. Gray lines depict the 95% CI. Fractional polynomial test is a goodness-of-fit test assessing whether any improvement of fit using a nonlinear function to model the data compared with a linear function is greater than expected due to chance alone.

**Figure 2.**
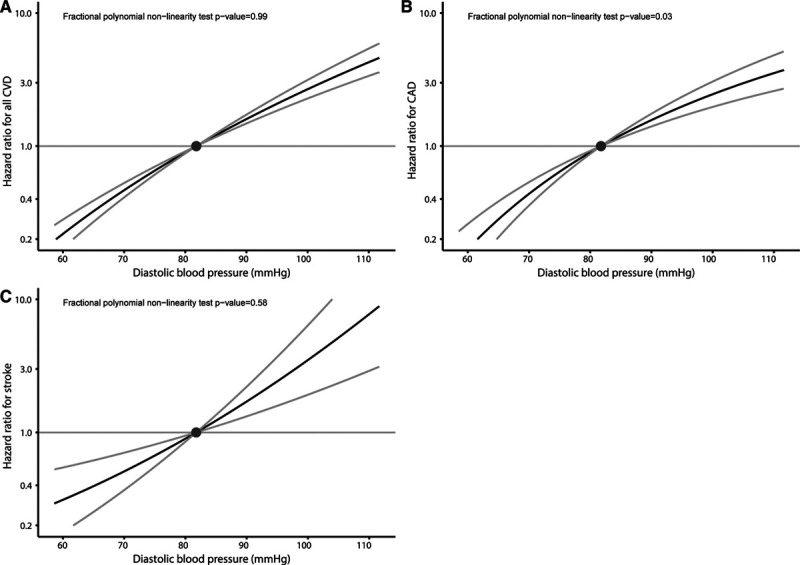
**Nonlinear Mendelian randomization considering genetically proxied diastolic blood pressure (DBP) and incident cardiovascular outcomes.** Nonlinear Mendelian randomization considering genetically proxied diastolic blood pressure (DBP) and incident cardiovascular outcomes: (**A**) all incident cardiovascular disease (CVD) events, (**B**) incident coronary artery disease (CAD), and (**C**) incident stroke. Displayed on the *x* axis are DBP values in mm Hg. The *y* axis shows the hazard ratio for the respective incident cardiovascular event. Reference is set to a population mean DBP value of 81.8 mm Hg. Gray lines depict the 95% CI.

While in some cases the best-fitting fractional polynomial was a nonlinear function, we observed no evidence favoring a nonlinear relationship between genetically proxied blood pressure and the cardiovascular outcomes over a linear one (Figures 1 and 2). This means that any departure from linearity was no greater than would be expected by chance due to random variability. Compared with the population mean SBP of 137 mm Hg, individuals with a genetically proxied SBP of 120 mm Hg had a 47% lower risk of incident CVD (HR, 0.53 [95% CI, 0.49–0.58]; Table 2). Compared with the population mean DBP of 82 mm Hg, individuals with a genetically proxied DBP of 70 mm Hg had a 53% lower risk of incident CVD (HR, 0.47 [95% CI, 0.41–0.53]; Table 2). MR estimates for population subgroups based on stratification into SBP and DBP centiles are provided in Tables S7 and S8, respectively.

**Table 2.**
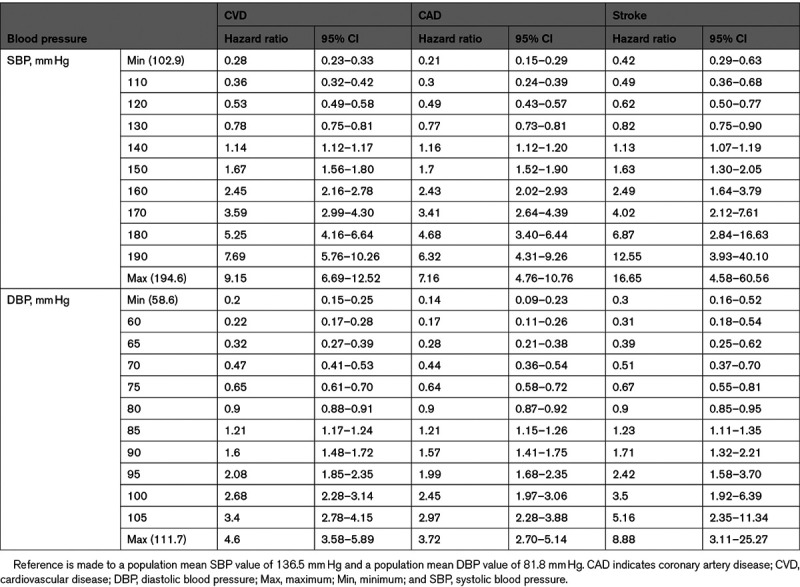
Nonlinear Mendelian Randomization Estimates for the Association Between SBP and Incident Cardiovascular Outcomes

**Figure 3.**
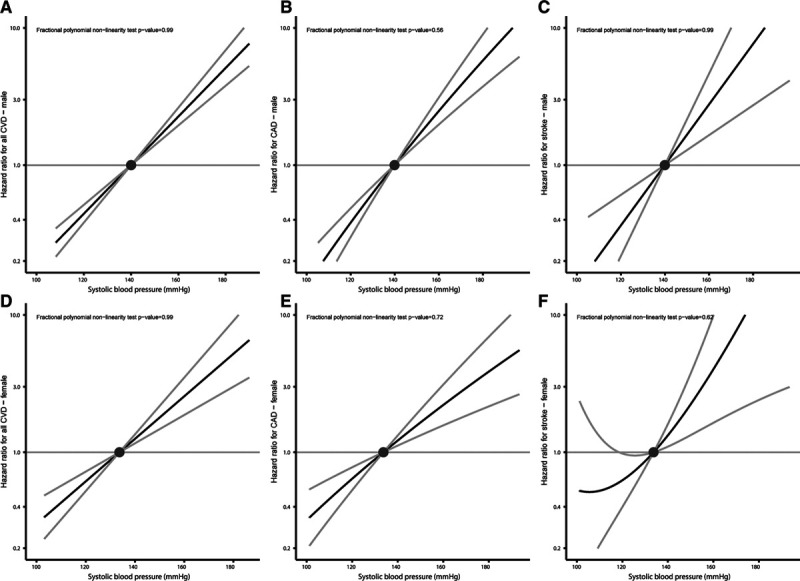
**Nonlinear Mendelian randomization considering genetically proxied systolic blood pressure (SBP) and incident cardiovascular outcomes split by sex.** Nonlinear Mendelian randomization considering genetically proxied systolic blood pressure (SBP) and incident cardiovascular outcomes split by sex: (**A**) all incident cardiovascular disease (CVD) events in men, (**B**) incident coronary artery disease (CAD) in males, and (**C**) incident stroke in men. **D–F**, Equivalent analyses in women. Displayed on the *x* axis are SBP values in mm Hg. The *y* axis shows the hazard ratio for the respective incident cardiovascular event. Reference is set to a mean SBP value of 136.5 mm Hg. Gray lines depict the 95% CI.

**Figure 4.**
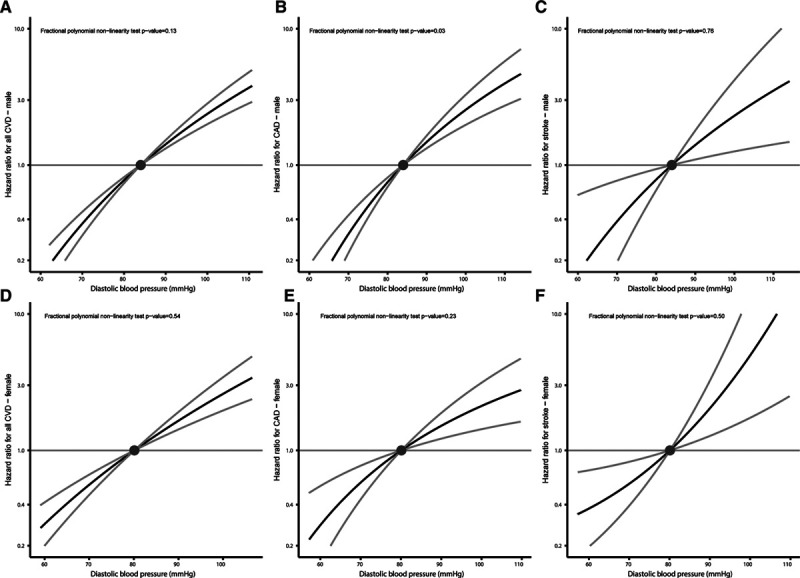
**Nonlinear Mendelian randomization considering genetically proxied diastolic blood pressure (DBP) and incident cardiovascular outcomes split by sex.** Nonlinear Mendelian randomization considering genetically proxied diastolic blood pressure (DBP) and incident cardiovascular outcomes split by sex: (**A**) all incident cardiovascular disease (CVD) events in men, (**B**) incident coronary artery disease (CAD) in men, and (**C**) incident stroke in men. **D–F**, Equivalent analyses in women. Displayed on the *x* axis are DBP values in mm Hg. The *y* axis shows the hazard ratio for the respective incident cardiovascular event. Reference is set to a mean DBP value of 81.8 mm Hg. Gray lines depict the 95% CI.

Subgroup analyses considering men and women separately produced similar results to the main analyses (Figures 3 and 4). Findings were also similar in the two sensitivity analyses: (1) using inverse probability weighting to correct for potential selection bias related to exclusion of individuals taking antihypertensive medications at baseline (Figures S3 and S4) and (2) using a different set of variants as instruments, which were obtained from studies not including the UK Biobank participants, and without adjustment for body mass index (Figures S5 and S6; and Table S9).

## Discussion

By applying nonlinear MR methods in the UK Biobank, we were able to examine the shape of the relationship between genetically proxied blood pressure and incident CVD in a population without a history of CVD or antihypertensive medication use. We found no evidence favoring nonlinear relationships between genetically proxied SBP or DBP and risk of the cardiovascular outcomes over linear ones. Similar results were obtained when considering males and females separately.

Blood pressure control represents a global health challenge,^33^ and hypertension thresholds have been lowered in recent consensus guidelines.^34^ The MR estimates obtained in this study may be used to quantify the effect of a persistent, lifelong reduction in blood pressure on the primary prevention of CVD and highlight the potential gains of clinical and public health interventions that achieve this. Importantly, they support the notion that for a population without a history of CVD or antihypertensive medication use, a similar relative reduction in CVD risk will be observed irrespective of baseline blood pressure, including for individuals who have normal blood pressure.^35^ This means that fixed changes in blood pressure will lead to similar changes in CVD risk on the HR scale. On the absolute scale, risk reduction will be greater for those with a higher baseline blood pressure. This finding is consistent with previous large-scale observational analyses performed in individuals free of CVD at baseline.^6,7^ In contrast, excessive blood pressure reduction in patients with atherosclerotic disease can reduce organ perfusion and increase CVD risk,^12^ and it is, therefore, important that our findings are not extrapolated to infer the effect of blood pressure lowering in individuals with preexisting CVD. It is also important to appreciate that absolute risk reduction conferred from blood pressure lowering will remain greatest for those with the highest blood pressure. Our current data support the concept that risk factor targeting in low- and medium-risk individuals on a population-wide level is likely to also substantially contribute to reducing the burden of CVD.^36,37^ Dietary modification and reduced sodium consumption represent examples of public health strategies that can be adopted to achieve this.^38,39^

We found no evidence for a J-shaped association of either genetically proxied SBP or DBP with any of the outcomes. This contrasts the findings of a recent observational study using data from 1.3 million general outpatients with a low prevalence of CAD,^8^ which identified a J-shaped association of blood pressure with the composite outcome of myocardial infarction and stroke. This J shape was only partially attenuated after adjusting for age, ethnicity, and comorbidities,^8^ and there remains the possibility that residual unknown or unmeasured confounding factors are responsible for the discrepancy with our findings. A systematic review and meta-analysis of blood pressure–lowering trials considering 613 815 participants from 123 studies found no trend for CVD risk reduction per 10 mm Hg lower SBP when stratifying trials by mean baseline SBP.^3^ In the SPRINT trial, SBP lowering to <120 mm Hg as compared with 140 mm Hg resulted in fewer major cardiovascular events.^11^ The findings from our current MR study additionally support a relative CVD risk reduction from blood pressure lowering below this level in patients without a history of CVD.

Our study has a number of strengths. By employing randomly allocated genetic variants as proxies for the effect of modifying blood pressure, we were able to use the MR paradigm to overcome the environmental confounding bias that can limit causal inference in observational association studies. The implementation of both linear and nonlinear MR methods within the comprehensive UK Biobank resource enabled us to efficiently study the relationships of genetically proxied SBP and DBP with incident CVD, CAD, and stroke, including in sex-stratified analyses. Importantly, the fractional polynomial method allowed us to investigate for evidence of nonlinear associations.

Our study also has limitations. This work only considered participants without a history of CVD or antihypertensive medication use, and its findings should not be extrapolated to populations with established CVD.^9,10^ Individuals that reported taking antihypertensive medications were excluded to allow for meaningful stratification into blood pressure quantiles, and as such, there is the possibility that ascertainment bias may have been introduced. Reassuringly, similar findings were obtained in inverse probability weighting sensitivity analyses, suggesting that any such bias is unlikely to be affecting our conclusions. The employed MR approach assumes that the genetic variants utilized as proxies for blood pressure do not affect CVD risk through alternative (pleiotropic) pathways—an assumption that cannot be tested and if violated could introduce bias to the obtained estimates. Our used MR method also explores the effects of lifelong changes in blood pressure, and its estimates should, therefore, not be extrapolated to quantify the effect of blood pressure modification in adult life, such as through use of antihypertensive medications. Finally, there were differences in the distribution of risk factors between individuals in the highest and lowest deciles of residual blood pressure (Table S5), suggesting that this MR analysis may still be vulnerable to environmental confounding.

## Perspectives

For a population without a history of CVD or antihypertensive medication use, genetically proxied blood pressure reduction was associated with lower CVD risk at all levels of blood pressure. These findings provide evidence to support that public health interventions achieving persistent, population-wide blood pressure reduction will be of considerable benefit in the primary prevention of CVD.

## Acknowledgments

This research has been conducted using the UK Biobank Resource (UK Biobank application No. 2532). The UK Biobank data are available on application at https://www.ukbiobank.ac.uk/register-apply. The Trøndelag Health Study is a collaboration between the HUNT Research Centre (Faculty of Medicine and Health Sciences, Norwegian University of Science and Technology), Nord-Trøndelag County Council, Central Norway Regional Health Authority, and the Norwegian Institute of Public Health. The genotype quality control and imputation in HUNT has been conducted by the K.G. Jebsen Center for Genetic Epidemiology, Department of Public Health and Nursing, Faculty of Medicine and Health Sciences, NTNU, Norwegian University of Science and Technology. D. Gill, R. Malik, M. Vujkovic, S. Burgess, and M.K. Georgakis designed the study. R. Malik, M. Vujkovic, J.N. Hellwege, M.M. Shuey, T.L. Edwards, B.M. Brumpton, T. Rogne, C. Fava, and A. Giontella analyzed the data. R. Malik, D. Gill, J.N. Hellwege, B.M. Brumpton, and M.K. Georgakis drafted the manuscript. All authors interpreted the results and critically revised the manuscript for intellectual content.

## Sources of Funding

This work was supported by the UK National Institute for Health Research Cambridge Biomedical Research Centre. M.K. Georgakis is funded by a scholarship from the Onassis Foundation. S.M. Damrauer was supported by the Department of Veterans Affairs Office of Research and Development (IK2-CX001780). P. Elliott acknowledges support from the British Heart Foundation (RE/18/4/34215), the Medical Research Council (MR/S019669/1), the National Institute for Health Research Imperial Biomedical Research Centre, Imperial College London (RDF03), the UK Dementia Research Institute (DRI) at Imperial College London funded by UK DRI, Ltd (funded by the Medical Research Council, Alzheimer’s Society, Alzheimer’s Research UK), and Health Data Research (HDR) UK London funded by HDR UK, Ltd (funded by a consortium led by the Medical Research Council 1004231). J.N. Hellwege is supported by K12 HD04348. M.M. Shuey is funded by the National Institutes of Health (DK108444). S. Burgess is supported by a Sir Henry Dale Fellowship jointly funded by the Wellcome Trust and the Royal Society (204623/Z/16/Z). M. Dichgans acknowledges funding from the European Union Horizon 2020 research and innovation programme (666881), SVDs@target (667375), CoSTREAM, SyNergy (EXC 2145 SyNergy, ID 390857198), the CRC 1123 (B3 and project DI 722/13-1), the Corona Foundation, the LMUexcellent Fond, the e:Med program (e:AtheroSysMed), and the FP7/2007-2103 European Union project CVgenes@target (Health-F2-2013-601456). D. Gill is supported by the Wellcome Trust 4i Programme (203928/Z/16/Z) and British Heart Foundation Centre of Research Excellence (RE/18/4/34215) at Imperial College London, and a National Institute for Health Research Clinical Lectureship at St. George’s, University of London (CL-2020-16-001). The BioVU dataset used for the analyses described was obtained from the Vanderbilt University Medical Center’s BioVU, which is supported by numerous sources: institutional funding, private agencies, and federal grants. These include the National Institutes of Health (NIH)–funded Shared Instrumentation Grant S10RR025141 and CTSA grants UL1TR002243, UL1TR000445, and UL1RR024975. Genomic data are also supported by investigator-led projects that include U01HG004798, R01NS032830, RC2GM092618, P50GM115305, U01HG006378, U19HL065962, and R01HD074711 and additional funding sources listed at https://victr.vumc.org/biovu-funding/. The genotyping in HUNT was financed by the NIH, University of Michigan, the Research Council of Norway; the Liaison Committee for Education, Research and Innovation in Central Norway, and the Joint Research Committee between St. Olav’s Hospital and the Faculty of Medicine and Health Sciences, NTNU.

## Disclosures

D. Gill is employed part-time by Novo Nordisk and has received consultancy fees from Abbott Laboratories. S.M. Damrauer has received grants from the US Department of Veterans Affairs, Calico Labs, and Renalytix AI plc outside the submitted work. The other authors report no conflicts.

## Supplementary Material



## Novelty and Significance

### What Is New?

- Recent methodological developments have enabled randomly allocated genetic variants to be leveraged in nonlinear Mendelian randomization analyses that explore the shape of the relationship between a risk factor and an outcome.
- Performing linear and nonlinear Mendelian randomization analyses in 255 714 European ancestry UK Biobank participants without a history of cardiovascular disease or antihypertensive medication use, this study found no evidence favoring nonlinear relationships of genetically proxied systolic and diastolic blood pressures with incident coronary artery disease, stroke, or a combined end point.

### What Is Relevant?

- At all levels of blood pressure, public health interventions achieving persistent blood pressure reduction are likely to be of considerable benefit in the primary prevention of cardiovascular disease.

### Summary

For a population without history of cardiovascular disease or antihypertensive medication use, genetically proxied blood pressure reduction was associated with lower cardiovascular disease risk at all levels of blood pressure.

## References

[1] NCD Risk Factor Collaboration. Worldwide trends in blood pressure from 1975 to 2015: a pooled analysis of 1479 population-based measurement studies with 19.1 million participants. Lancet. 2017;389:37–55. doi: 10.1016/S0140-6736(16)31919-5 27863813 10.1016/S0140-6736(16)31919-5 PMC5220163

[2] Yusuf S Joseph P Rangarajan S Islam S Mente A Hystad P Brauer M Kutty VR Gupta R Wielgosz A. Modifiable risk factors, cardiovascular disease, and mortality in 155 722 individuals from 21 high-income, middle-income, and low-income countries (PURE): a prospective cohort study. Lancet. 2020;395:795–808. doi: 10.1016/S0140-6736(19)32008-2 31492503 10.1016/S0140-6736(19)32008-2 PMC8006904

[3] Ettehad D Emdin CA Kiran A Anderson SG Callender T Emberson J Chalmers J Rodgers A Rahimi K. Blood pressure lowering for prevention of cardiovascular disease and death: a systematic review and meta-analysis. Lancet. 2016;387:957–967. doi: 10.1016/S0140-6736(15)01225-8 26724178 10.1016/S0140-6736(15)01225-8

[4] Arnett DK Blumenthal RS Albert MA Buroker AB Goldberger ZD Hahn EJ Himmelfarb CD Khera A Lloyd-Jones D McEvoy JW. 2019 ACC/AHA guideline on the primary prevention of cardiovascular disease: executive summary: a report of the American College of Cardiology/American Heart Association Task Force on Clinical Practice Guidelines. Circulation. 2019;140:e563–e595. doi: 10.1161/CIR.0000000000000677 30879339 10.1161/CIR.0000000000000677 PMC8351755

[5] Piepoli MF Hoes AW Agewall S Albus C Brotons C Catapano AL Cooney MT Corrà U Cosyns B Deaton C; ESC Scientific Document Group. 2016 European Guidelines on cardiovascular disease prevention in clinical practice: the Sixth Joint Task Force of the European Society of Cardiology and other Societies on Cardiovascular Disease Prevention in Clinical Practice (constituted by representatives of 10 societies and by invited experts)Developed with the special contribution of the European Association for Cardiovascular Prevention & Rehabilitation (EACPR). Eur Heart J. 2016;37:2315–2381. doi: 10.1093/eurheartj/ehw106 27222591 10.1093/eurheartj/ehw106 PMC4986030

[6] Rapsomaniki E Timmis A George J Pujades-Rodriguez M Shah AD Denaxas S White IR Caulfield MJ Deanfield JE Smeeth L. Blood pressure and incidence of twelve cardiovascular diseases: lifetime risks, healthy life-years lost, and age-specific associations in 1·25 million people. Lancet. 2014;383:1899–1911. doi: 10.1016/S0140-6736(14)60685-1 24881994 10.1016/S0140-6736(14)60685-1 PMC4042017

[7] Lewington S Clarke R Qizilbash N Peto R Collins R; Prospective Studies Collaboration. Age-specific relevance of usual blood pressure to vascular mortality: a meta-analysis of individual data for one million adults in 61 prospective studies. Lancet. 2002;360:1903–1913. doi: 10.1016/s0140-6736(02)11911-8 12493255 10.1016/s0140-6736(02)11911-8

[8] Flint AC Conell C Ren X Banki NM Chan SL Rao VA Melles RB Bhatt DL. Effect of systolic and diastolic blood pressure on cardiovascular outcomes. N Engl J Med. 2019;381:243–251. doi: 10.1056/NEJMoa1803180 31314968 10.1056/NEJMoa1803180

[9] Vidal-Petiot E Ford I Greenlaw N Ferrari R Fox KM Tardif JC Tendera M Tavazzi L Bhatt DL Steg PG; CLARIFY Investigators. Cardiovascular event rates and mortality according to achieved systolic and diastolic blood pressure in patients with stable coronary artery disease: an international cohort study. Lancet. 2016;388:2142–2152. doi: 10.1016/S0140-6736(16)31326-5 27590221 10.1016/S0140-6736(16)31326-5

[10] Ovbiagele B Diener HC Yusuf S Martin RH Cotton D Vinisko R Donnan GA Bath PM; PROFESS Investigators. Level of systolic blood pressure within the normal range and risk of recurrent stroke. JAMA. 2011;306:2137–2144. doi: 10.1001/jama.2011.1650 22089721 10.1001/jama.2011.1650

[11] Wright JT Jr Williamson JD Whelton PK Snyder JK Sink KM Rocco MV Reboussin DM Rahman M Oparil S Lewis CE; Sprint Research Group. A randomized trial of intensive versus standard blood-pressure control. N Engl J Med. 2015;373:2103–16. 26551272 10.1056/NEJMoa1511939 PMC4689591

[12] Mancia G Grassi G. Aggressive blood pressure lowering is dangerous: the J-curve: pro side of the arguement. Hypertension. 2014;63:29–36. doi: 10.1161/01.hyp.0000441190.09494.e9 24336629 10.1161/01.hyp.0000441190.09494.e9

[13] Nazarzadeh M Pinho-Gomes AC Smith Byrne K Canoy D Raimondi F Ayala Solares JR Otto CM Rahimi K. Systolic blood pressure and risk of valvular heart disease: a Mendelian randomization study. JAMA Cardiol. 2019;4:788–795. doi: 10.1001/jamacardio.2019.2202 31290937 10.1001/jamacardio.2019.2202 PMC6624812

[14] Burgess S Butterworth A Malarstig A Thompson SG. Use of Mendelian randomisation to assess potential benefit of clinical intervention. BMJ. 2012;345:e7325. doi: 10.1136/bmj.e7325 23131671 10.1136/bmj.e7325

[15] Sun YQ Burgess S Staley JR Wood AM Bell S Kaptoge SK Guo Q Bolton TR Mason AM Butterworth AS. Body mass index and all cause mortality in HUNT and UK Biobank studies: linear and non-linear mendelian randomisation analyses. BMJ. 2019;364:l1042. doi: 10.1136/bmj.l1042 30957776 10.1136/bmj.l1042 PMC6434515

[16] Staley JR Burgess S. Semiparametric methods for estimation of a nonlinear exposure-outcome relationship using instrumental variables with application to Mendelian randomization. Genet Epidemiol. 2017;41:341–352. doi: 10.1002/gepi.22041 28317167 10.1002/gepi.22041 PMC5400068

[17] Burgess S Davies NM Thompson SG; EPIC-InterAct Consortium. Instrumental variable analysis with a nonlinear exposure-outcome relationship. Epidemiology. 2014;25:877–885. doi: 10.1097/EDE.0000000000000161 25166881 10.1097/EDE.0000000000000161 PMC4222800

[18] Davey Smith G Davies NM Dimou N Egger M Gallo V Golub R. STROBE-MR: guidelines for strengthening the reporting of Mendelian randomization studies. PeerJ Preprints. Preprint posted online July 15, 2019

[19] Bycroft C Freeman C Petkova D Band G Elliott LT Sharp K Motyer A Vukcevic D Delaneau O O’Connell J. The UK Biobank resource with deep phenotyping and genomic data. Nature. 2018;562:203–209. doi: 10.1038/s41586-018-0579-z 30305743 10.1038/s41586-018-0579-z PMC6786975

[20] Evangelou E Warren HR Mosen-Ansorena D Mifsud B Pazoki R Gao H Ntritsos G Dimou N Cabrera CP Karaman I; Million Veteran Program. Genetic analysis of over 1 million people identifies 535 new loci associated with blood pressure traits. Nat Genet. 2018;50:1412–1425. doi: 10.1038/s41588-018-0205-x 30224653 10.1038/s41588-018-0205-x PMC6284793

[21] Holmes MV Davey Smith G. Problems in interpreting and using GWAS of conditional phenotypes illustrated by ‘alcohol GWAS’. Mol Psychiatry. 2019;24:167–168. doi: 10.1038/s41380-018-0037-1 29520038 10.1038/s41380-018-0037-1 PMC6004313

[22] Willer CJ Li Y Abecasis GR. METAL: fast and efficient meta-analysis of genomewide association scans. Bioinformatics. 2010;26:2190–2191. doi: 10.1093/bioinformatics/btq340 20616382 10.1093/bioinformatics/btq340 PMC2922887

[23] Purcell S Neale B Todd-Brown K Thomas L Ferreira MA Bender D Maller J Sklar P de Bakker PI Daly MJ. PLINK: a tool set for whole-genome association and population-based linkage analyses. Am J Hum Genet. 2007;81:559–575. doi: 10.1086/519795 17701901 10.1086/519795 PMC1950838

[24] Burgess S Dudbridge F Thompson SG. Combining information on multiple instrumental variables in Mendelian randomization: comparison of allele score and summarized data methods. Stat Med. 2016;35:1880–1906. doi: 10.1002/sim.6835 26661904 10.1002/sim.6835 PMC4832315

[25] Burgess S Small DS Thompson SG. A review of instrumental variable estimators for Mendelian randomization. Stat Methods Med Res. 2017;26:2333–2355. doi: 10.1177/0962280215597579 26282889 10.1177/0962280215597579 PMC5642006

[26] Burgess S Thompson SG. Use of allele scores as instrumental variables for Mendelian randomization. Int J Epidemiol. 2013;42:1134–1144. doi: 10.1093/ije/dyt093 24062299 10.1093/ije/dyt093 PMC3780999

[27] Palmer TM Lawlor DA Harbord RM Sheehan NA Tobias JH Timpson NJ Davey Smith G Sterne JA. Using multiple genetic variants as instrumental variables for modifiable risk factors. Stat Methods Med Res. 2012;21:223–242. doi: 10.1177/0962280210394459 21216802 10.1177/0962280210394459 PMC3917707

[28] Slob EAW Burgess S. A comparison of robust Mendelian randomization methods using summary data. Genet Epidemiol. 2020;44:313–329. doi: 10.1002/gepi.22295 32249995 10.1002/gepi.22295 PMC7317850

[29] Bowden J Davey Smith G Burgess S. Mendelian randomization with invalid instruments: effect estimation and bias detection through Egger regression. Int J Epidemiol. 2015;44:512–525. doi: 10.1093/ije/dyv080 26050253 10.1093/ije/dyv080 PMC4469799

[30] Verbanck M Chen CY Neale B Do R. Detection of widespread horizontal pleiotropy in causal relationships inferred from Mendelian randomization between complex traits and diseases. Nat Genet. 2018;50:693–698. doi: 10.1038/s41588-018-0099-7 29686387 10.1038/s41588-018-0099-7 PMC6083837

[31] Didelez V Sheehan N. Mendelian randomization as an instrumental variable approach to causal inference. Stat Methods Med Res. 2007;16:309–330. doi: 10.1177/0962280206077743 17715159 10.1177/0962280206077743

[32] Wootton RE Richmond RC Stuijfzand BG Lawn RB Sallis HM Taylor GMJ Hemani G Jones HJ Zammit S Smith GD. Evidence for causal effects of lifetime smoking on risk for depression and schizophrenia: a Mendelian randomisation study. Psychol Med. 2019;50:1–9. 31689377 10.1017/S0033291719002678 PMC7610182

[33] Chow CK Gupta R. Blood pressure control: a challenge to global health systems. Lancet. 2019;394:613–615. doi: 10.1016/S0140-6736(19)31293-0 31327567 10.1016/S0140-6736(19)31293-0

[34] Whelton PK Carey RM Aronow WS Casey DE Jr Collins KJ Dennison Himmelfarb C DePalma SM Gidding S Jamerson KA Jones DW. 2017 ACC/AHA/AAPA/ABC/ACPM/AGS/APhA/ASH/ASPC/NMA/PCNA guideline for the prevention, detection, evaluation, and management of high blood pressure in adults: executive summary: a report of the American College of Cardiology/American Heart Association Task Force on Clinical Practice Guidelines. Hypertension. 2018;71:1269–1324. doi: 10.1161/HYP.0000000000000066 29133354 10.1161/HYP.0000000000000066

[35] Rose G. Sick individuals and sick populations. Int J Epidemiol. 1985;14:32–38. doi: 10.1093/ije/14.1.32 3872850 10.1093/ije/14.1.32

[36] Emberson J Whincup P Morris R Walker M Ebrahim S. Evaluating the impact of population and high-risk strategies for the primary prevention of cardiovascular disease. Eur Heart J. 2004;25:484–491. doi: 10.1016/j.ehj.2003.11.012 15039128 10.1016/j.ehj.2003.11.012

[37] Hardy ST Loehr LR Butler KR Chakladar S Chang PP Folsom AR Heiss G MacLehose RF Matsushita K Avery CL. Reducing the blood pressure-related burden of cardiovascular disease: impact of achievable improvements in blood pressure prevention and control. J Am Heart Assoc. 2015;4:e002276. doi: 10.1161/JAHA.115.002276 26508742 10.1161/JAHA.115.002276 PMC4845128

[38] He FJ Brinsden HC MacGregor GA. Salt reduction in the United Kingdom: a successful experiment in public health. J Hum Hypertens. 2014;28:345–352. doi: 10.1038/jhh.2013.105 24172290 10.1038/jhh.2013.105

[39] Xu A Ma J Guo X Wang L Wu J Zhang J Bai Y Xu J Lu Z Xu Z. Association of a province-wide intervention with salt intake and hypertension in shandong province, China, 2011-2016. JAMA Intern Med. 2020;180:877–886. doi: 10.1001/jamainternmed.2020.0904 32338717 10.1001/jamainternmed.2020.0904 PMC7186913

