# Supplementary figures and images for: Relationship Between Blood Pressure and Incident Cardiovascular Disease: Linear and Nonlinear Mendelian Randomization Analyses
Source: Hypertension. 2021 Apr 5;77(6):2004–13. doi: 10.1161/HYPERTENSIONAHA.120.16534 (PMC8115430; doi:10.1161/HYPERTENSIONAHA.120.16534)

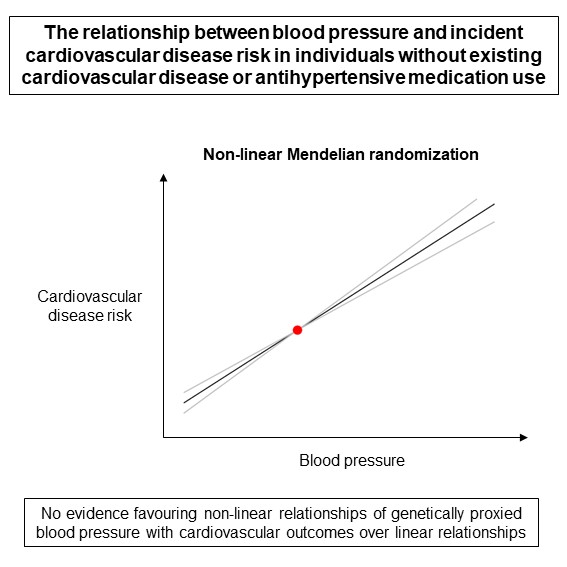

Supplement: Supplementary file 1 [file hyp-77-2004-s001.jpg]
