# Supplementary material for: Relationship Between Blood Pressure and Incident Cardiovascular Disease: Linear and Nonlinear Mendelian Randomization Analyses
Source: Hypertension. 2021 Apr 5;77(6):2004–13. doi: 10.1161/HYPERTENSIONAHA.120.16534 (PMC8115430; doi:10.1161/HYPERTENSIONAHA.120.16534)
Supplement: Supplementary file 2 [file hyp-77-2004-s002.docx]

**Title page**

**ONLINE SUPPLEMENT**

**The relationship between blood pressure and incident cardiovascular disease: linear and non-linear Mendelian randomization analyses**

**Corresponding author**

Dr Dipender Gill, Department of Epidemiology and Biostatistics, School of Public Health, Medical School Building, St Mary's Hospital, Imperial College London, United Kingdom, W2 1PG, Telephone: +44 (0) 7904843810, Fax: +44 (0) 20 3313 8223, E-mail: [dipender.gill@imperial.ac.uk](mailto:dipender.gill@imperial.ac.uk)

**CONTENTS**

[**Supplemental Text** 3](#_Toc62208930)

[**Supplemental References** 14](#_Toc62208931)

[**Table S1.** 16](#_Toc62208932)

[**Table S2.** 17](#_Toc62208933)

[**Table S3.** 25](#_Toc62208934)

[**Table S4.** 26](#_Toc62208935)

[**Table S5** 27](#_Toc62208936)

[**Table S6.** 28](#_Toc62208937)

[**Table S7.** 29](#_Toc62208938)

[**Table S8.** 31](#_Toc62208939)

[**Table S9.** 33](#_Toc62208940)

[**Figure S1.** 34](#_Toc62208941)

[**Figure S2.** 35](#_Toc62208942)

[**Figure S3.** 36](#_Toc62208943)

[**Figure S4.** 37](#_Toc62208944)

[**Figure S5.** 38](#_Toc62208945)

[**Figure S6.** 39](#_Toc62208946)

**Supplemental Text**

**Supplementary Methods**

***Instruments that did not adjust for body mass index***

In the main Mendelian randomization (MR) analysis, genetic variants to proxy blood pressure modification were selected from a study of 299,024 participants (not included in the UK Biobank) that adjusted for body mass index (1). As this could theoretically introduce collider bias in MR analyses (2), we performed sensitivity analyses where genetic variants modelled as instruments were selected from analyses that did not adjust for body mass index. Two cohorts were used for this – the BioVU DNA repository (N=53,285) and the Nord-Trøndelag Health Study (N=69,076), which are further detailed below. To pool genetic association estimates from the two studies, fixed-effects meta-analysis was performed using METAL (22). To select genetic instruments, variants reaching genome-wide significance (p<5x10^-8^) in the meta-analysis were clumped to correlation r^2^<0.01 using PLINK (23). For systolic blood pressure (SBP) and diastolic blood pressure (DBP), we extracted 22 and 27 uncorrelated variants respectively (**Supplementary Tables 3 and 4**).

*BioVU DNA Repository*

The BioVU DNA Repository is a deidentified database of electronic health records (EHR) that are linked to patient DNA samples at Vanderbilt University Medical Center. A detailed description of the database and how it is maintained has been published elsewhere (3). BioVU participant DNA samples were genotyped on a custom Illumina Multi-Ethnic Genotyping Array (MEGA-ex; Illumina Inc., San Diego, CA, USA). Quality control included excluding samples or variants with missingness rates above 2%. Samples were also excluded if consent had been revoked, sample was duplicated, or failed sex concordance checks. Imputation was performed on the Michigan Imputation Server (MIS) v1.2.4 (4), using Minimac4 and the Haplotype Reference Consortium (HRC) panel v1.1 (5).

Among BioVU participants, we selected unrelated self-reported White adults (age ≥ 18) and used the earliest median eligible non-Emergency Department outpatient measured SBP in the EHR, and the corresponding DBP. Measures were considered ineligible if they occurred at or after an International Classification of Diseases (ICD)-9/10 billing code from the groups 585/N18, 405/I15, or 428/I50. For measures taken while a patient was on an antihypertensive medication we added 15 mmHg to SBP and 10 mmHg to DBP.

We performed linear regression association tests with additive models for untransformed blood pressure traits, after correcting for medication use. We adjusted linear regression models analyzing single-nucleotide polymorphism (SNP) associations for age at blood pressure measure, age^2^, sex, and the top 10 genetic principal components of genetic ancestry using SNPTEST-v2.5.4-beta26. Inferences were limited to genotyped and imputed variants with imputation info scores of 0.4 or higher, Hardy-Weinberg equilibrium p-values >5x10^-8^, and minor allele frequencies >0.01. Data from a total of 53,285 participants were included in analyses.

*Nord-Trøndelag Health Study*

The Nord-Trøndelag Health Study (HUNT) is a population-based health survey conducted in the county of Nord Trøndelag, Norway. Individuals were included at four different time points during approximately 20 years (HUNT1 [1984-1986], HUNT2 [1995-1997], HUNT3 [2006-2008]) and HUNT4 [2017-2019]) (6). At each time point, the entire adult population (≥ 20 years) was invited to participate by completing questionnaires, attending clinical examinations and interviews. Participation rates have generally been high: 89.4% (n = 77,212), 69.5% (n = 65,237), 54.1% (n = 50,807) and 54.0% (n=56,042) in HUNT1, HUNT2, HUNT3 and HUNT4, respectively (6). Taken together, the health studies include information from over 120,000 different individuals from Nord-Trøndelag. Biological samples including DNA have been collected for approximately 90,000 participants. SBP and DBP were measured three times (in mmHg) with one minute intervals, with the person seated, using a Dinamap 845 XT and XL9301 (Critikon, Tampa, FL) oscillometer in HUNT2 and a 8100 and XL9301 in HUNT3. We used the mean of the second and third measurements, and if a third measurement was not conducted (12% of measurements in HUNT3), only the second measurement was used. Cuff size was adjusted to the participant’s arm circumference. 15mmHg and 10 mmHg was added to the SBP and DBP measurements, respectively, if the participant answered “Yes” to “Are you taking medication for high blood pressure?” at HUNT2 or “Do you take, or have you taken medication for high blood pressure?” at HUNT3 at the time of measurement. Measurements from HUNT2 were prioritized. The HumanCoreExome-12 v 1.0 (Illumina), HumanCoreExome12 v1.1 (Illumina) and UM HUNT Biobank v1.0 was used to genotype approximately 600,000 variants. Genotype calling was performed with Genome Studio (Illumina). Samples with genotype call rates <99% were excluded, as were those with gender mismatches, duplicates, had contamination > 2.5% as estimated with BAF Regress and evidence of non-European ancestry from principal components analysis (p-value < 0.0001). Variants were excluded if the cluster separation score was <0.3, Gentrain score was >0.15, genotyping fail rate >1%, or they deviated from Hardy-Weinberg equilibrium (P <1x10^-4^). Samples were phased with Eagle2 v2.3 (7), and genotype imputation was conducted with Minimac3 (v2.0.1, http://genome.sph.umich.edu/wiki/Minimac3) (4), and a merged reference panel that was constructed by combining the Haplotype Reference Consortium panel (release version 1.1) (5) and a local reference panel based on 2,202 whole genome sequenced HUNT study participants. Associations with blood pressure were tested using a generalized mixed model including covariates age, age^2^, sex, genotype batch, and principal components 1-4 of genetic ancestry as implemented in BOLT (version 2.3.4) (8). Principal components were computed using PLINK. Additional filters applied to the analysis included minor allele count ≥ 10 and imputation r^2^≥ 0.3. Data from a total of 69,076 participants were included in analyses.

***Non-linear MR***

*Fractional polynomial method*

The fractional polynomial method in MR is a flexible parametric method devised to find the best-fitting model (linear or non-linear) for the causal relationship between an exposure and outcome. A full description of the method is provided by Staley and Burgess (9). We outline the method below.

Fractional polynomials are a family of functions introduced by Royston and Altman (10). We consider first-order and second-order fractional polynomials. A first-order fractional polynomial is of the form:

$$f\left( x \right)=\beta_{0}+\beta_{1}x^{p}$$

and a second-order polynomial is of the form:

$$f\left( x \right)=\left\{ \begin{aligned} \beta_{0}+\beta_{1}x^{p_{1}}+\beta_{2}x^{p_{2}}, &\text{if} p_{1}\neq p_{2} \\ \beta_{0}+\beta_{1}x^{p}+\beta_{2}x^{p}log(x), &\text{if} p=p_{1}=p_{2} \end{aligned} \right.$$

where $p,p_{1},p_{2}\in\{ -2, -1, -0.5, 0, 0.5, 1, 2, 3\}$ and the power of 0 refers to the (natural) log function. These are the standard powers recommended by Royston and Altman (10).

When performing the fractional polynomial method, linear MR estimates obtained in each centile of residual blood pressure (referred to as ‘localized average causal effects’) are meta-regressed against a function of the mean blood pressure in that centile. This function is the derivative of a fractional polynomial. We perform meta-regression for derivatives of all first- and second-degree fractional polynomials, and take as the best fitting model the fractional polynomial with the maximum value of the log-likelihood amongst all fractional polynomials, where the log-likelihood is obtained from the meta-regression model. These are the functions plotted in Figures 1-4.

*Fractional polynomial test*

The fractional polynomial test compares whether a non-linear fractional polynomial model fits the data better than a linear model, and specifically tests whether any improvement in fit over the linear model is greater than would be expected by chance due to random variability. We note that even if the true relationship between the exposure and outcome is linear, a non-linear model is more flexible and so could fit the observed data better due to chance alone.

We calculate the test statistic as twice the difference in the log-likelihood between the best-fitting fractional polynomial of degree 1 and the linear model, and compare this test statistic to a chi-squared distribution with 1 degree of freedom. The null hypothesis is that the true causal relationship between the exposure and outcome is linear. A low p-value for the fractional polynomial test indicates that the improvement in fit with the best-fitting fractional polynomial of degree 1 compared to the fit with the linear model is more extreme than would be expected due to chance alone, indicating that a non-linear model is favoured over a linear model.

***Inverse-probability weighting sensitivity analysis***

Individuals with elevated blood pressure are more likely to be prescribed antihypertensive medications, and therefore exclusion of these individuals from the main analysis could potentially distort non-linear MR estimates due to introduction of collider bias. Inverse-probability weighting was therefore performed in a sensitivity analysis to investigate this. First, we modelled antihypertensive drug use with age, sex, body mass index, lifetime smoking index, deprivation index quintiles, low-density lipoprotein cholesterol levels, diabetes diagnosis and assessment centre as explanatory variables in a logistic regression performed on all individuals with available data in the UK Biobank. After exclusion of individuals on antihypertensive medication, the fitted values from the first model were taken as standardized inverse probability weights in the Cox proportional hazards model used to generate association estimates for the allele score with outcomes. Individuals with a high risk factor similarity to individuals on antihypertensive drugs were thus counted more often in the Cox proportional hazards model to attenuate any potential effect of selection bias. Weights were trimmed at the 99^th^ centile.

**Supplementary Checklist – STROBE-MR Reporting Guidelines**

1. TITLE and ABSTRACT

Indicate Mendelian randomization as the study’s design in the title and/or the abstract.

***Detailed in the Title and Abstract sections.***

INTRODUCTION

2. Background

Explain the scientific background and rationale for the reported study. Is causality between exposure and outcome plausible? Justify why MR is a helpful method to address the study question.

***Detailed in the Background section.***

3. Objectives

State specific objectives clearly, including pre-specified causal hypotheses (if any).

***Detailed in the Background section.***

METHODS

4. Study design and data sources

Present key elements of study design early in the paper. Consider including a table listing sources of data for all phases of the study. For each data source contributing to the analysis, describe the following:

a) Describe the study design and the underlying population from which it was drawn.

Describe also the setting, locations, and relevant dates, including periods of recruitment, exposure, follow-up, and data collection, if available.

b) Give the eligibility criteria, and the sources and methods of selection of participants.

c) Explain how the analyzed sample size was arrived at.

d) Describe measurement, quality and selection of genetic variants.

e) For each exposure, outcome and other relevant variables, describe methods of assessment and, in the case of diseases, the diagnostic criteria used.

f) Provide details of ethics committee approval and participant informed consent, if relevant.

***Detailed in the Methods and Supplementary Methods sections.***

5. Assumptions

Explicitly state assumptions for the main analysis (e.g. relevance, exclusion, independence, homogeneity) as well assumptions for any additional or sensitivity analysis.

***Detailed in the Methods and Supplementary Methods sections.***

6. Statistical methods: main analysis

Describe statistical methods and statistics used.

a) Describe how quantitative variables were handled in the analyses (i.e., scale, units, model).

b) Describe the process for identifying genetic variants and weights to be included in the analyses (i.e, independence and model). Consider a flow diagram.

c) Describe the MR estimator, e.g. two-stage least squares, Wald ratio, and related statistics.

Detail the included covariates and, in case of two-sample MR, whether the same covariate set was used for adjustment in the two samples.

d) Explain how missing data were addressed.

e) If applicable, say how multiple testing was dealt with.

***Detailed in the Methods and Supplementary Methods sections.***

7. Assessment of assumptions

Describe any methods used to assess the assumptions or justify their validity.

***Detailed in the Methods, Supplementary Methods and Discussion sections.***

8. Sensitivity analyses

Describe any sensitivity analyses or additional analyses performed.

***Detailed in the Methods and Supplementary Methods sections.***

9. Software and pre-registration

a) Name statistical software and package(s), including version and settings used.

***Detailed in the Methods and Supplementary Methods sections.***

b) State whether the study protocol and details were pre-registered (as well as when and where).

***Detailed in the Methods sections.***

RESULTS

10. Descriptive data

a) Report the numbers of individuals at each stage of included studies and reasons for exclusion. Consider use of a flow-diagram.

b) Report summary statistics for phenotypic exposure(s), outcome(s) and other relevant variables (e.g. means, standard deviations, proportions).

c) If the data sources include meta-analyses of previous studies, provide the number of studies, their reported ancestry, if available, and assessments of heterogeneity across these studies. Consider using a supplementary table for each data source.

d) For two-sample Mendelian randomization:

i. Provide information on the similarity of the genetic variant-exposure associations between the exposure and outcome samples.

ii. Provide information on extent of sample overlap between the exposure and outcome data sources.

***Detailed in the Methods, Supplementary Methods, Results and Supplementary Tables sections.***

11. Main results

a) Report the associations between genetic variant and exposure, and between genetic variant and outcome, preferably on an interpretable scale (e.g. comparing 25th and 75th percentile of allele count or genetic risk score, if individual-level data available).

b) Report causal effect estimate between exposure and outcome, and the measures of uncertainty from the MR analysis. Use an intuitive scale, such as odds ratio, or relative risk, per standard deviation difference.

c) If relevant, consider translating estimates of relative risk into absolute risk for a meaningful time-period.

d) Consider any plots to visualize results (e.g. forest plot, scatterplot of associations between genetic variants and outcome versus between genetic variants and exposure).

***Detailed in the Results, Supplementary Tables and Supplementary Figures sections.***

12. Assessment of assumptions

a) Assess the validity of the assumptions.

b) Report any additional statistics (e.g., assessments of heterogeneity, such as I2, Q statistic).

***Detailed in the Results, Supplementary Tables and Discussion sections.***

13. Sensitivity and additional analyses

a) Use sensitivity analyses to assess the robustness of the main results to violations of the assumptions.

b) Report results from other sensitivity analyses (e.g., replication study with different dataset, analyses of subgroups, validation of instrument(s), simulations, etc.).

c) Report any assessment of direction of causality (e.g., bidirectional MR).

d) When relevant, report and compare with estimates from non-MR analyses.

e) Consider any additional plots to visualize results (e.g., leave-one-out analyses).

***Detailed in the Results, Supplementary Tables and Supplementary Figures sections.***

DISCUSSION

14. Key results

Summarize key results with reference to study objectives.

***Detailed in the Discussion section.***

15. Limitations

Discuss limitations of the study, taking into account the validity of the MR assumptions, other sources of potential bias, and imprecision. Discuss both direction and magnitude of any potential bias, and any efforts to address them.

***Detailed in the Discussion section.***

16. Interpretation

a) Give a cautious overall interpretation of results considering objectives and limitations.

Compare with results from other relevant studies.

b) Discuss underlying biological mechanisms that could be modelled by using the genetic variants to assess the relationship between the exposure and the outcome.

c) Discuss whether the results have clinical or policy relevance, and whether interventions could have the same size effect.

***Detailed in the Discussion section.***

17. Generalizability

Discuss the generalizability of the study results (a) to other populations (i.e. external validity),

(b) across other exposure periods/timings, and (c) across other levels of exposure.

***Detailed in the Discussion section.***

OTHER INFORMATION

18. Funding

Give the source of funding and the role of the funders for the present study and, if applicable, for the original study or studies on which the present article is based.

***Detailed in the Funding section.***

19. Data and data sharing

Present data used to perform all analyses or report where and how the data can be accessed. State whether statistical code is publicly accessible and if so, where.

***Detailed in the Methods section.***

20. Conflicts of Interest

All authors should declare all potential conflicts of interest.

***Detailed in the Disclosures section.***

# **Supplemental References**

1. Evangelou E, Warren HR, Mosen-Ansorena D, Mifsud B, Pazoki R, Gao H, et al. Genetic analysis of over 1 million people identifies 535 new loci associated with blood pressure traits. Nat Genet. 2018:50(10):1412-25.

2. Holmes MV, Davey Smith G. Problems in interpreting and using GWAS of conditional phenotypes illustrated by 'alcohol GWAS'. Mol Psychiatry. 2018.

3. Roden DM, Pulley JM, Basford MA, Bernard GR, Clayton EW, Balser JR, et al. Development of a large-scale de-identified DNA biobank to enable personalized medicine. Clin Pharmacol Ther. 2008;84(3):362-9.

4. Das S, Forer L, Schonherr S, Sidore C, Locke AE, Kwong A, et al. Next-generation genotype imputation service and methods. Nat Genet. 2016;48(10):1284-7.

5. McCarthy S, Das S, Kretzschmar W, Delaneau O, Wood AR, Teumer A, et al. A reference panel of 64,976 haplotypes for genotype imputation. Nat Genet. 2016;48(10):1279-83.

6. Krokstad S, Langhammer A, Hveem K, Holmen TL, Midthjell K, Stene TR, et al. Cohort Profile: the HUNT Study, Norway. Int J Epidemiol. 2013;42(4):968-77.

7. Loh PR, Danecek P, Palamara PF, Fuchsberger C, Reshef YA, Finucane HK, et al. Reference-based phasing using the Haplotype Reference Consortium panel. Nat Genet. 2016;48(11):1443-8.

8. Loh PR, Tucker G, Bulik-Sullivan BK, Vilhjalmsson BJ, Finucane HK, Salem RM, et al. Efficient Bayesian mixed-model analysis increases association power in large cohorts. Nat Genet. 2015;47(3):284-90.

9. Staley JR, Burgess S. Semiparametric methods for estimation of a nonlinear exposure-outcome relationship using instrumental variables with application to Mendelian randomization. Genet Epidemiol. 2017;41(4):341-52.

10. Royston P, Altman DG. Regression Using Fractional Polynomials of Continuous Covariates - Parsimonious Parametric Modeling. J R Stat Soc C-Appl. 1994;43(3):429-67.

**Table S1.** Hospitalization-based International Diagnostic Classification of Diseases and Health Related Problems version 10 (ICD-10) and Office of Population Censuses and Surveys (OPCS) Classification of Surgical Operations and Procedures (4th revision, OPCS-4) codes to identify incident coronary artery disease (CAD) and stroke events.

| CAD | ICD-10 | I10-I25 |
| --- | --- | --- |
|  | OPCS-4 | K40-K46, K49, K50 and K75 |
| stroke | ICD-10 | I60,I60.0,I60.1 I60.2, I60.3, I60.4, I60.5, I60.6, I60.7, I60.8, I60.9, I61, I61.0, I61.1, I61.2, I61.3, I61.4, I61.5, I61.6, I61.8, I61.9, I63, I63.0, I63.2, I63.3, I63.4, I63.5, I63.6, I63.8, I63.9, I64.* |

* indicates that all sub-codes are included.

**Table S2.** The genetic variant used in the main analyses as genetic proxies for systolic blood pressure (SBP) and diastolic blood pressure (DBP). The association estimates were taken from the International Consortium of Blood Pressure study (N=299,024), published in Supplementary Table 24 of Evangelou et al 2018 (1), and did not include UK Biobank participants.

| **RSID** | **Chromosome** | **Position** | **Effect allele** | **Other allele** | **Effect allele frequency** | **Beta SBP (mmHg)** | **SE SBP (mmHg)** | **P SBP** | **Beta DBP (mmHg)** | **SE DBP (mmHg)** | **P DBP** | Remarks |
| --- | --- | --- | --- | --- | --- | --- | --- | --- | --- | --- | --- | --- |
| rs880315 | 1 | 10796866 | T | C | 0.652 | -0.522 | 0.050 | 1.33E-25 | -0.252 | 0.029 | 6.79E-18 |  |
| rs17030613 | 1 | 113190807 | A | C | 0.781 | -0.392 | 0.056 | 2.45E-12 | -0.236 | 0.033 | 8.53E-13 | Not available in UKB |
| rs2932538 | 1 | 113216543 | A | G | 0.259 | -0.344 | 0.053 | 7.15E-11 | -0.237 | 0.031 | 2.31E-14 | Not available in UKB |
| rs17367504 | 1 | 11862778 | A | G | 0.844 | 0.777 | 0.064 | 4.81E-34 | 0.472 | 0.038 | 6.22E-36 |  |
| rs3820068 | 1 | 15798197 | A | G | 0.798 | 0.336 | 0.060 | 1.69E-08 | 0.128 | 0.035 | 2.46E-04 |  |
| rs2076328 | 1 | 1687482 | T | G | 0.475 | -0.282 | 0.050 | 1.42E-08 | -0.097 | 0.029 | 9.29E-04 |  |
| rs2157597 | 1 | 169201567 | T | C | 0.347 | -0.086 | 0.049 | 8.06E-02 | 0.003 | 0.029 | 9.05E-01 | Excluded in MR-PRESSO (CAD-SBP) |
| rs12405515 | 1 | 172357441 | T | G | 0.574 | -0.171 | 0.046 | 2.04E-04 | -0.162 | 0.027 | 2.64E-09 |  |
| rs4245739 | 1 | 204518842 | A | C | 0.733 | 0.170 | 0.053 | 1.18E-03 | 0.136 | 0.031 | 9.99E-06 |  |
| rs2761436 | 1 | 207919748 | T | C | 0.528 | 0.193 | 0.046 | 2.88E-05 | 0.031 | 0.027 | 2.51E-01 |  |
| rs12408022 | 1 | 217718789 | T | C | 0.261 | 0.251 | 0.053 | 2.39E-06 | 0.149 | 0.031 | 2.05E-06 |  |
| rs73091767 | 1 | 227250775 | T | C | 0.735 | -0.091 | 0.052 | 7.95E-02 | -0.087 | 0.030 | 4.05E-03 |  |
| rs2004776 | 1 | 230848702 | T | C | 0.238 | 0.337 | 0.054 | 5.33E-10 | 0.236 | 0.032 | 1.47E-13 |  |
| rs6429422 | 1 | 243472801 | T | G | 0.671 | -0.143 | 0.049 | 3.60E-03 | -0.192 | 0.029 | 3.62E-11 |  |
| rs6686889 | 1 | 25030470 | T | C | 0.258 | 0.085 | 0.053 | 1.08E-01 | 0.115 | 0.031 | 2.30E-04 |  |
| rs2493292 | 1 | 3328659 | T | C | 0.150 | 0.269 | 0.068 | 7.77E-05 | 0.167 | 0.040 | 3.01E-05 |  |
| rs9729719 | 1 | 38298207 | A | G | 0.295 | 0.102 | 0.053 | 5.55E-02 | -0.034 | 0.031 | 2.69E-01 |  |
| rs7515635 | 1 | 42408070 | T | C | 0.468 | 0.238 | 0.046 | 2.70E-07 | 0.109 | 0.027 | 6.52E-05 |  |
| rs147696085 | 1 | 51021867 | A | G | 0.101 | -0.074 | 0.080 | 3.54E-01 | 0.142 | 0.047 | 2.56E-03 |  |
| rs112557609 | 1 | 56576924 | A | G | 0.341 | 0.238 | 0.049 | 1.08E-06 | 0.083 | 0.029 | 3.87E-03 |  |
| rs2404715 | 1 | 57008778 | T | C | 0.093 | -0.200 | 0.080 | 1.22E-02 | 0.002 | 0.047 | 9.73E-01 | Excluded in MR-PRESSO (CAD-SBP) |
| rs60199046 | 1 | 59663341 | A | G | 0.713 | 0.178 | 0.051 | 4.35E-04 | -0.076 | 0.030 | 1.08E-02 |  |
| rs709209 | 1 | 6278414 | A | G | 0.658 | 0.094 | 0.053 | 7.64E-02 | -0.027 | 0.031 | 3.79E-01 |  |
| rs10922502 | 1 | 89360158 | A | G | 0.641 | -0.228 | 0.048 | 2.32E-06 | -0.105 | 0.028 | 2.20E-04 | Not available in UKB |
| rs9662255 | 1 | 9441949 | A | C | 0.431 | -0.188 | 0.048 | 9.41E-05 | -0.016 | 0.028 | 5.80E-01 |  |
| rs112184198 | 10 | 102604514 | A | G | 0.106 | -0.533 | 0.076 | 2.40E-12 | -0.300 | 0.045 | 1.95E-11 |  |
| rs11191548 | 10 | 104846178 | T | C | 0.913 | 1.023 | 0.082 | 6.19E-36 | 0.455 | 0.048 | 4.08E-21 |  |
| rs4387287 | 10 | 105677897 | A | C | 0.174 | 0.195 | 0.062 | 1.76E-03 | 0.167 | 0.037 | 5.60E-06 |  |
| rs34872471 | 10 | 114754071 | T | C | 0.706 | -0.180 | 0.051 | 4.22E-04 | -0.007 | 0.030 | 8.25E-01 |  |
| rs2782980 | 10 | 115781527 | T | C | 0.290 | -0.339 | 0.052 | 7.64E-11 | -0.242 | 0.031 | 2.63E-15 |  |
| rs4373814 | 10 | 18419972 | C | G | 0.441 | 0.252 | 0.047 | 6.21E-08 | 0.160 | 0.027 | 6.21E-09 | Excluded in linear MR due to being palindromic with intermediate allele frequencies |
| rs1813353 | 10 | 18707448 | T | C | 0.659 | 0.432 | 0.048 | 3.00E-19 | 0.277 | 0.028 | 1.70E-22 |  |
| rs9337951 | 10 | 30317073 | A | G | 0.337 | 0.093 | 0.053 | 7.85E-02 | -0.113 | 0.031 | 2.77E-04 | Excluded in MR-PRESSO (CAD-SBP) |
| rs11008355 | 10 | 31412561 | C | G | 0.237 | -0.086 | 0.054 | 1.15E-01 | 0.033 | 0.032 | 3.00E-01 |  |
| rs10826995 | 10 | 32082658 | T | C | 0.721 | -0.090 | 0.051 | 8.14E-02 | 0.021 | 0.030 | 4.93E-01 |  |
| rs2246438 | 10 | 45273079 | A | G | 0.278 | -0.022 | 0.051 | 6.72E-01 | -0.071 | 0.030 | 1.77E-02 |  |
| rs1530440 | 10 | 63524591 | T | C | 0.187 | -0.498 | 0.059 | 1.96E-17 | -0.359 | 0.035 | 2.45E-25 |  |
| rs7914287 | 10 | 69350563 | T | C | 0.770 | -0.149 | 0.056 | 7.84E-03 | -0.002 | 0.033 | 9.58E-01 |  |
| rs932764 | 10 | 95895940 | A | G | 0.556 | -0.365 | 0.047 | 4.84E-15 | -0.148 | 0.028 | 7.18E-08 |  |
| rs4494250 | 10 | 96563757 | A | G | 0.361 | 0.256 | 0.049 | 1.33E-07 | 0.177 | 0.029 | 6.25E-10 |  |
| rs633185 | 11 | 100593538 | C | G | 0.710 | 0.498 | 0.051 | 9.80E-23 | 0.280 | 0.030 | 7.49E-21 | Excluded in MR-PRESSO (CAD-SBP) |
| rs7129220 | 11 | 10350538 | A | G | 0.123 | 0.392 | 0.072 | 6.28E-08 | 0.189 | 0.043 | 9.36E-06 |  |
| rs8258 | 11 | 117283676 | T | C | 0.368 | 0.056 | 0.048 | 2.43E-01 | -0.087 | 0.028 | 2.05E-03 |  |
| rs900145 | 11 | 13293905 | T | C | 0.704 | 0.114 | 0.050 | 2.30E-02 | 0.119 | 0.029 | 5.00E-05 |  |
| rs4757391 | 11 | 16302939 | T | C | 0.801 | -0.492 | 0.057 | 8.30E-18 | -0.300 | 0.034 | 5.40E-19 |  |
| rs381815 | 11 | 16902268 | T | C | 0.271 | 0.364 | 0.052 | 1.45E-12 | 0.204 | 0.030 | 1.44E-11 |  |
| rs5219 | 11 | 17409572 | T | C | 0.376 | 0.320 | 0.047 | 1.12E-11 | 0.158 | 0.028 | 1.32E-08 |  |
| rs661348 | 11 | 1905292 | T | C | 0.563 | -0.342 | 0.050 | 9.56E-12 | -0.161 | 0.029 | 3.74E-08 |  |
| rs11030119 | 11 | 27728102 | A | G | 0.294 | -0.170 | 0.051 | 8.50E-04 | -0.133 | 0.030 | 8.68E-06 |  |
| rs7480089 | 11 | 45207851 | A | G | 0.122 | -0.233 | 0.072 | 1.11E-03 | -0.015 | 0.042 | 7.30E-01 |  |
| rs7103648 | 11 | 47461783 | A | G | 0.616 | -0.310 | 0.047 | 6.16E-11 | -0.188 | 0.028 | 1.75E-11 |  |
| rs11537751 | 11 | 47587452 | T | C | 0.052 | 0.394 | 0.108 | 2.56E-04 | 0.211 | 0.063 | 7.54E-04 |  |
| rs11229457 | 11 | 58207203 | T | C | 0.214 | -0.289 | 0.056 | 2.97E-07 | -0.159 | 0.033 | 1.71E-06 |  |
| rs751984 | 11 | 61278246 | T | C | 0.879 | 0.434 | 0.074 | 4.06E-09 | 0.363 | 0.043 | 6.25E-17 |  |
| rs3741378 | 11 | 65408937 | T | C | 0.133 | -0.417 | 0.070 | 2.15E-09 | -0.182 | 0.041 | 8.37E-06 |  |
| rs67330701 | 11 | 69079707 | T | C | 0.096 | -0.277 | 0.091 | 2.42E-03 | -0.231 | 0.053 | 1.50E-05 |  |
| rs7927515 | 11 | 76125330 | A | C | 0.346 | 0.171 | 0.049 | 4.79E-04 | 0.091 | 0.029 | 1.58E-03 |  |
| rs110419 | 11 | 8252853 | A | G | 0.493 | 0.081 | 0.047 | 8.53E-02 | 0.117 | 0.028 | 2.36E-05 |  |
| rs7126805 | 11 | 828916 | A | G | 0.729 | 0.120 | 0.056 | 3.14E-02 | -0.010 | 0.033 | 7.61E-01 |  |
| rs2289125 | 11 | 89224453 | A | C | 0.230 | -0.194 | 0.058 | 8.45E-04 | 0.079 | 0.034 | 2.12E-02 |  |
| rs3184504 | 12 | 111884608 | T | C | 0.476 | 0.577 | 0.047 | 2.57E-35 | 0.441 | 0.028 | 9.98E-57 | Excluded in MR-PRESSO (CAD-SBP) |
| rs10850411 | 12 | 115387796 | T | C | 0.699 | 0.304 | 0.049 | 8.06E-10 | 0.186 | 0.030 | 3.16E-10 |  |
| rs35444 | 12 | 115552437 | A | G | 0.608 | 0.332 | 0.047 | 1.79E-12 | 0.220 | 0.028 | 5.54E-15 |  |
| rs11067763 | 12 | 116198341 | A | G | 0.897 | 0.191 | 0.075 | 1.13E-02 | 0.143 | 0.045 | 1.51E-03 |  |
| rs1060105 | 12 | 123806219 | T | C | 0.202 | -0.101 | 0.057 | 7.85E-02 | -0.164 | 0.034 | 1.57E-06 |  |
| rs12579720 | 12 | 20173764 | C | G | 0.238 | -0.302 | 0.054 | 2.52E-08 | -0.271 | 0.032 | 5.98E-17 |  |
| rs6487543 | 12 | 26438189 | A | G | 0.766 | 0.175 | 0.056 | 1.86E-03 | 0.106 | 0.034 | 1.64E-03 |  |
| rs1126930 | 12 | 49399132 | C | G | 0.034 | 0.576 | 0.140 | 3.93E-05 | 0.260 | 0.083 | 1.73E-03 |  |
| rs7977389 | 12 | 49981722 | T | C | 0.894 | 0.164 | 0.075 | 2.77E-02 | 0.080 | 0.045 | 7.23E-02 |  |
| rs7302981 | 12 | 50537815 | A | G | 0.387 | 0.375 | 0.046 | 4.39E-16 | 0.231 | 0.028 | 5.69E-17 |  |
| rs73099903 | 12 | 53440779 | T | C | 0.079 | 0.422 | 0.088 | 1.56E-06 | 0.219 | 0.052 | 2.75E-05 |  |
| rs7297416 | 12 | 54443090 | A | C | 0.687 | 0.282 | 0.050 | 1.84E-08 | 0.130 | 0.030 | 1.41E-05 |  |
| rs17249754 | 12 | 90060586 | A | G | 0.164 | -0.802 | 0.062 | 2.16E-38 | -0.408 | 0.037 | 3.50E-28 | Excluded in MR-PRESSO (CAD-SBP) |
| rs76785029 | 12 | 94882905 | T | C | 0.072 | -0.079 | 0.097 | 4.15E-01 | 0.168 | 0.058 | 3.77E-03 |  |
| rs9549328 | 13 | 113636156 | T | C | 0.235 | 0.217 | 0.055 | 8.55E-05 | 0.071 | 0.033 | 3.15E-02 |  |
| rs63418562 | 13 | 30146201 | T | C | 0.746 | -0.385 | 0.053 | 3.74E-13 | -0.215 | 0.032 | 1.08E-11 | Not available in UKB |
| rs1475130 | 14 | 100225144 | T | C | 0.343 | -0.164 | 0.049 | 7.39E-04 | 0.000 | 0.029 | 9.97E-01 |  |
| rs452036 | 14 | 23865885 | A | G | 0.349 | -0.144 | 0.048 | 2.72E-03 | 0.086 | 0.029 | 2.64E-03 |  |
| rs8904 | 14 | 35871217 | A | G | 0.374 | 0.320 | 0.048 | 2.00E-11 | 0.120 | 0.029 | 2.43E-05 |  |
| rs9888615 | 14 | 53377540 | T | C | 0.294 | -0.236 | 0.050 | 2.32E-06 | -0.081 | 0.030 | 6.63E-03 |  |
| rs9323988 | 14 | 98587630 | T | C | 0.610 | -0.238 | 0.046 | 3.05E-07 | -0.111 | 0.028 | 6.78E-05 |  |
| rs1036477 | 15 | 48914926 | A | G | 0.896 | 0.340 | 0.074 | 4.12E-06 | -0.066 | 0.044 | 1.38E-01 |  |
| rs956006 | 15 | 62808539 | T | C | 0.336 | -0.070 | 0.049 | 1.57E-01 | 0.043 | 0.030 | 1.50E-01 |  |
| rs7178615 | 15 | 66869072 | A | G | 0.392 | -0.141 | 0.047 | 2.80E-03 | -0.133 | 0.028 | 2.71E-06 |  |
| rs1378942 | 15 | 75077367 | A | C | 0.656 | -0.487 | 0.048 | 5.00E-24 | -0.373 | 0.029 | 3.53E-38 |  |
| rs62011052 | 15 | 79156983 | T | C | 0.850 | -0.017 | 0.064 | 7.98E-01 | 0.153 | 0.039 | 6.99E-05 |  |
| rs2759308 | 15 | 81016227 | A | G | 0.476 | 0.259 | 0.046 | 1.79E-08 | 0.118 | 0.028 | 1.90E-05 |  |
| rs2034618 | 15 | 83799632 | T | C | 0.223 | -0.015 | 0.055 | 7.83E-01 | -0.109 | 0.033 | 8.81E-04 |  |
| rs2521501 | 15 | 91437388 | A | T | 0.670 | -0.557 | 0.052 | 4.78E-27 | -0.353 | 0.031 | 2.10E-30 | Excluded in MR-PRESSO (CAD-SBP) |
| rs12906962 | 15 | 95312071 | T | C | 0.671 | -0.266 | 0.049 | 6.44E-08 | -0.188 | 0.030 | 1.70E-10 |  |
| rs13333226 | 16 | 20365654 | A | G | 0.814 | 0.336 | 0.058 | 6.96E-09 | 0.253 | 0.035 | 3.07E-13 |  |
| rs11639856 | 16 | 24788645 | A | T | 0.190 | -0.229 | 0.058 | 6.81E-05 | -0.068 | 0.034 | 4.70E-02 |  |
| rs72799341 | 16 | 30936743 | A | G | 0.239 | 0.095 | 0.054 | 7.90E-02 | 0.157 | 0.032 | 1.12E-06 |  |
| rs12921187 | 16 | 4943019 | T | G | 0.427 | -0.242 | 0.046 | 1.27E-07 | -0.156 | 0.027 | 1.22E-08 |  |
| rs33063 | 16 | 69640217 | A | G | 0.144 | 0.178 | 0.065 | 6.02E-03 | -0.069 | 0.039 | 7.41E-02 |  |
| rs35261357 | 16 | 75444572 | T | C | 0.570 | 0.278 | 0.047 | 2.68E-09 | 0.103 | 0.028 | 2.42E-04 |  |
| rs8059962 | 16 | 81574197 | T | C | 0.424 | -0.169 | 0.047 | 3.01E-04 | -0.114 | 0.028 | 4.54E-05 |  |
| rs7500448 | 16 | 83045790 | A | G | 0.747 | 0.225 | 0.053 | 2.40E-05 | -0.026 | 0.032 | 4.12E-01 | Excluded in MR-PRESSO (CAD-SBP) |
| rs1126464 | 16 | 89704365 | C | G | 0.245 | 0.185 | 0.057 | 1.08E-03 | 0.222 | 0.034 | 4.74E-11 |  |
| rs12941318 | 17 | 1333598 | T | C | 0.498 | -0.208 | 0.048 | 1.71E-05 | -0.080 | 0.029 | 5.52E-03 |  |
| rs79089478 | 17 | 40317241 | T | C | 0.972 | 0.140 | 0.146 | 3.39E-01 | -0.066 | 0.087 | 4.51E-01 |  |
| rs12946454 | 17 | 43208121 | A | T | 0.739 | -0.319 | 0.052 | 7.30E-10 | -0.150 | 0.031 | 1.24E-06 |  |
| rs115231027 | 17 | 44199290 | T | C | 0.824 | -0.257 | 0.070 | 2.31E-04 | -0.109 | 0.041 | 7.75E-03 | Not available in UKB |
| rs17608766 | 17 | 45013271 | T | C | 0.857 | -0.615 | 0.067 | 3.89E-20 | -0.189 | 0.040 | 2.21E-06 |  |
| rs7406910 | 17 | 46688256 | T | C | 0.089 | -0.488 | 0.081 | 1.93E-09 | -0.159 | 0.049 | 1.02E-03 |  |
| rs12940887 | 17 | 47402807 | T | C | 0.373 | 0.266 | 0.047 | 1.42E-08 | 0.248 | 0.028 | 9.73E-19 | Excluded in MR-PRESSO (CAD-SBP) |
| rs2645466 | 17 | 57853214 | A | C | 0.698 | -0.196 | 0.049 | 7.05E-05 | -0.030 | 0.030 | 3.04E-01 |  |
| rs2240736 | 17 | 59485393 | T | C | 0.733 | 0.427 | 0.053 | 4.49E-16 | 0.248 | 0.031 | 2.83E-15 |  |
| rs740698 | 17 | 60767151 | T | C | 0.573 | -0.146 | 0.048 | 2.24E-03 | 0.016 | 0.029 | 5.67E-01 |  |
| rs4308 | 17 | 61559625 | A | G | 0.382 | 0.273 | 0.048 | 1.08E-08 | 0.183 | 0.029 | 1.34E-10 |  |
| rs28427409 | 17 | 6473882 | T | C | 0.413 | 0.164 | 0.046 | 3.98E-04 | -0.003 | 0.028 | 9.26E-01 |  |
| rs2467099 | 17 | 73949045 | T | C | 0.220 | -0.191 | 0.055 | 4.79E-04 | -0.084 | 0.033 | 1.08E-02 |  |
| rs57927100 | 17 | 75317300 | C | G | 0.741 | 0.348 | 0.054 | 8.43E-11 | 0.238 | 0.032 | 9.95E-14 |  |
| rs78378222 | 17 | 7571752 | T | G | 0.984 | 0.183 | 0.208 | 3.79E-01 | -0.613 | 0.124 | 7.44E-07 |  |
| rs12958173 | 18 | 42141977 | A | C | 0.300 | 0.352 | 0.050 | 1.21E-12 | 0.177 | 0.030 | 2.04E-09 |  |
| rs7236548 | 18 | 43097750 | A | C | 0.186 | 0.264 | 0.058 | 5.53E-06 | 0.005 | 0.035 | 8.92E-01 |  |
| rs745821 | 18 | 48142854 | T | G | 0.749 | 0.177 | 0.053 | 7.52E-04 | 0.141 | 0.032 | 7.44E-06 |  |
| rs36010659 | 18 | 48283949 | T | C | 0.861 | 0.195 | 0.066 | 3.01E-03 | -0.005 | 0.039 | 9.04E-01 |  |
| rs167479 | 19 | 11526765 | T | G | 0.473 | -0.428 | 0.056 | 2.50E-14 | -0.342 | 0.034 | 1.06E-23 |  |
| rs17638167 | 19 | 11584818 | T | C | 0.047 | -0.523 | 0.110 | 1.81E-06 | -0.238 | 0.066 | 2.89E-04 |  |
| rs10418305 | 19 | 15278808 | C | G | 0.103 | -0.078 | 0.075 | 3.02E-01 | 0.086 | 0.045 | 5.76E-02 |  |
| rs2304130 | 19 | 19789528 | A | G | 0.917 | -0.307 | 0.086 | 3.57E-04 | -0.234 | 0.051 | 5.14E-06 |  |
| rs740406 | 19 | 2232221 | A | G | 0.937 | -0.549 | 0.102 | 6.90E-08 | -0.199 | 0.060 | 1.00E-03 |  |
| rs62104477 | 19 | 30294991 | T | G | 0.331 | 0.087 | 0.049 | 7.41E-02 | 0.130 | 0.029 | 7.93E-06 |  |
| rs8105753 | 19 | 31927547 | A | C | 0.626 | 0.190 | 0.049 | 9.88E-05 | 0.092 | 0.029 | 1.61E-03 |  |
| rs7248104 | 19 | 7224431 | A | G | 0.409 | -0.159 | 0.046 | 5.96E-04 | -0.040 | 0.028 | 1.47E-01 |  |
| rs4247374 | 19 | 7252756 | T | C | 0.136 | -0.506 | 0.075 | 1.76E-11 | -0.339 | 0.045 | 5.38E-14 |  |
| rs1438896 | 2 | 145646072 | T | C | 0.300 | 0.228 | 0.050 | 5.39E-06 | 0.191 | 0.029 | 8.25E-11 |  |
| rs1446468 | 2 | 164963486 | T | C | 0.451 | -0.487 | 0.047 | 2.26E-25 | -0.263 | 0.028 | 1.51E-21 |  |
| rs79146658 | 2 | 179786068 | T | C | 0.918 | -0.005 | 0.086 | 9.58E-01 | -0.282 | 0.051 | 2.36E-08 |  |
| rs16823124 | 2 | 183224127 | A | G | 0.303 | 0.232 | 0.050 | 3.13E-06 | 0.180 | 0.029 | 6.87E-10 |  |
| rs7592578 | 2 | 191439591 | T | G | 0.207 | -0.341 | 0.060 | 1.47E-08 | -0.188 | 0.035 | 1.20E-07 |  |
| rs1344653 | 2 | 19730845 | A | G | 0.496 | -0.157 | 0.046 | 5.80E-04 | 0.068 | 0.027 | 1.13E-02 |  |
| rs55780018 | 2 | 208526140 | T | C | 0.548 | -0.328 | 0.049 | 1.85E-11 | -0.144 | 0.029 | 5.02E-07 |  |
| rs7255 | 2 | 20878820 | T | C | 0.466 | -0.071 | 0.047 | 1.31E-01 | 0.075 | 0.028 | 6.38E-03 |  |
| rs1063281 | 2 | 218668732 | T | C | 0.605 | -0.239 | 0.048 | 7.13E-07 | -0.138 | 0.028 | 1.12E-06 |  |
| rs2972146 | 2 | 227100698 | T | G | 0.636 | 0.249 | 0.048 | 1.76E-07 | 0.140 | 0.028 | 6.56E-07 |  |
| rs55701159 | 2 | 25139596 | T | G | 0.887 | 0.300 | 0.074 | 5.27E-05 | 0.147 | 0.044 | 7.51E-04 |  |
| rs1275988 | 2 | 26914364 | T | C | 0.606 | -0.516 | 0.047 | 1.83E-28 | -0.279 | 0.027 | 2.48E-24 |  |
| rs9678851 | 2 | 27887034 | A | C | 0.559 | -0.114 | 0.047 | 1.66E-02 | -0.059 | 0.028 | 3.55E-02 |  |
| rs7562 | 2 | 28635740 | T | C | 0.530 | 0.156 | 0.047 | 9.30E-04 | 0.083 | 0.028 | 2.57E-03 |  |
| rs13420463 | 2 | 37517566 | A | G | 0.778 | 0.275 | 0.056 | 7.19E-07 | 0.127 | 0.033 | 9.46E-05 |  |
| rs4952611 | 2 | 40567743 | T | C | 0.583 | -0.214 | 0.049 | 1.19E-05 | -0.134 | 0.029 | 2.64E-06 |  |
| rs76326501 | 2 | 43167878 | A | C | 0.911 | 0.551 | 0.083 | 3.05E-11 | 0.360 | 0.049 | 1.39E-13 |  |
| rs11690961 | 2 | 46363336 | A | C | 0.884 | 0.023 | 0.072 | 7.53E-01 | -0.166 | 0.042 | 9.64E-05 |  |
| rs1975487 | 2 | 55809054 | A | G | 0.488 | -0.221 | 0.047 | 2.93E-06 | -0.174 | 0.028 | 3.75E-10 |  |
| rs3771371 | 2 | 71627539 | T | C | 0.566 | -0.181 | 0.046 | 9.15E-05 | -0.068 | 0.027 | 1.22E-02 |  |
| rs1876487 | 2 | 73114352 | A | C | 0.288 | -0.083 | 0.055 | 1.30E-01 | -0.111 | 0.032 | 5.36E-04 |  |
| rs11689667 | 2 | 85491365 | T | C | 0.546 | 0.119 | 0.047 | 1.05E-02 | -0.035 | 0.027 | 2.04E-01 |  |
| rs2579519 | 2 | 96675166 | T | C | 0.605 | -0.005 | 0.048 | 9.11E-01 | -0.150 | 0.028 | 1.35E-07 |  |
| rs1327235 | 20 | 10969030 | A | G | 0.537 | -0.355 | 0.045 | 3.20E-15 | -0.239 | 0.027 | 5.98E-19 |  |
| rs6081613 | 20 | 19465907 | A | G | 0.272 | 0.136 | 0.051 | 7.27E-03 | -0.088 | 0.030 | 3.55E-03 |  |
| rs6060114 | 20 | 30169673 | T | C | 0.838 | 0.281 | 0.062 | 5.91E-06 | 0.162 | 0.037 | 1.25E-05 |  |
| rs6031435 | 20 | 42797358 | A | G | 0.539 | -0.227 | 0.046 | 6.72E-07 | -0.111 | 0.027 | 4.74E-05 |  |
| rs6095241 | 20 | 47308798 | A | G | 0.436 | -0.188 | 0.045 | 3.30E-05 | -0.124 | 0.027 | 5.04E-06 |  |
| rs6015450 | 20 | 57751117 | A | G | 0.871 | -0.655 | 0.069 | 2.44E-21 | -0.480 | 0.041 | 2.85E-31 |  |
| rs6108168 | 20 | 8626271 | A | C | 0.254 | -0.204 | 0.052 | 8.84E-05 | -0.142 | 0.031 | 4.97E-06 |  |
| rs11701033 | 21 | 33788341 | C | G | 0.817 | -0.247 | 0.059 | 3.18E-05 | -0.112 | 0.035 | 1.59E-03 |  |
| rs12627651 | 21 | 44760603 | A | G | 0.294 | 0.416 | 0.053 | 3.32E-15 | 0.248 | 0.032 | 4.20E-15 |  |
| rs9306160 | 21 | 45107562 | T | C | 0.397 | -0.207 | 0.047 | 1.29E-05 | -0.169 | 0.028 | 2.82E-09 |  |
| rs12628032 | 22 | 19967980 | T | C | 0.315 | 0.197 | 0.050 | 7.70E-05 | 0.018 | 0.030 | 5.47E-01 |  |
| rs4823006 | 22 | 29451671 | A | G | 0.562 | 0.141 | 0.046 | 1.98E-03 | 0.110 | 0.027 | 5.37E-05 |  |
| rs470113 | 22 | 40729614 | A | G | 0.812 | -0.078 | 0.058 | 1.76E-01 | 0.060 | 0.035 | 8.37E-02 |  |
| rs73161324 | 22 | 42038786 | T | C | 0.059 | 0.212 | 0.116 | 6.66E-02 | -0.112 | 0.069 | 1.05E-01 |  |
| rs347591 | 3 | 11290122 | T | G | 0.663 | 0.284 | 0.049 | 6.31E-09 | 0.147 | 0.029 | 3.33E-07 |  |
| rs62270945 | 3 | 128201889 | T | C | 0.030 | 0.158 | 0.158 | 3.19E-01 | -0.221 | 0.091 | 1.55E-02 |  |
| rs6783086 | 3 | 133959552 | T | C | 0.409 | 0.257 | 0.047 | 3.93E-08 | 0.146 | 0.028 | 1.11E-07 |  |
| rs2306374 | 3 | 138119952 | T | C | 0.838 | -0.227 | 0.063 | 3.04E-04 | -0.170 | 0.037 | 4.38E-06 | Excluded in MR-PRESSO (CAD-SBP) |
| rs16851397 | 3 | 141134818 | A | G | 0.953 | -0.591 | 0.114 | 1.94E-07 | -0.400 | 0.066 | 1.74E-09 |  |
| rs11128722 | 3 | 14958126 | A | G | 0.563 | -0.252 | 0.047 | 8.53E-08 | -0.119 | 0.028 | 1.83E-05 |  |
| rs143112823 | 3 | 154707967 | A | G | 0.076 | -0.402 | 0.095 | 2.29E-05 | -0.178 | 0.055 | 1.23E-03 |  |
| rs419076 | 3 | 169100886 | T | C | 0.474 | 0.395 | 0.046 | 5.38E-18 | 0.250 | 0.027 | 1.38E-20 |  |
| rs12374077 | 3 | 185317674 | C | G | 0.345 | 0.170 | 0.048 | 4.57E-04 | 0.126 | 0.029 | 9.64E-06 |  |
| rs13082711 | 3 | 27537909 | T | C | 0.769 | -0.281 | 0.055 | 2.78E-07 | -0.184 | 0.032 | 1.03E-08 |  |
| rs9815354 | 3 | 41912651 | A | G | 0.171 | -0.063 | 0.063 | 3.18E-01 | 0.326 | 0.037 | 1.33E-18 |  |
| rs6797587 | 3 | 48197614 | A | G | 0.320 | -0.280 | 0.050 | 1.50E-08 | -0.221 | 0.029 | 3.03E-14 |  |
| rs36022378 | 3 | 49913705 | T | C | 0.807 | -0.156 | 0.060 | 9.30E-03 | -0.114 | 0.035 | 1.19E-03 |  |
| rs13303 | 3 | 52558008 | T | C | 0.430 | -0.110 | 0.047 | 2.07E-02 | -0.015 | 0.028 | 5.82E-01 |  |
| rs9810888 | 3 | 53635595 | T | G | 0.498 | -0.151 | 0.046 | 1.15E-03 | -0.128 | 0.027 | 2.74E-06 |  |
| rs9827472 | 3 | 56726646 | T | C | 0.358 | -0.164 | 0.049 | 7.23E-04 | -0.143 | 0.029 | 5.63E-07 |  |
| rs918466 | 3 | 64710253 | A | G | 0.411 | -0.139 | 0.048 | 3.62E-03 | -0.164 | 0.028 | 5.52E-09 |  |
| rs13107325 | 4 | 103188709 | T | C | 0.072 | -0.878 | 0.095 | 1.79E-20 | -0.604 | 0.056 | 1.50E-27 |  |
| rs13112725 | 4 | 106911742 | C | G | 0.768 | 0.397 | 0.056 | 1.01E-12 | 0.214 | 0.033 | 6.70E-11 |  |
| rs6825911 | 4 | 111381638 | T | C | 0.787 | -0.238 | 0.058 | 3.73E-05 | -0.171 | 0.034 | 4.73E-07 |  |
| rs66887589 | 4 | 120509279 | T | C | 0.519 | -0.181 | 0.046 | 8.98E-05 | -0.125 | 0.027 | 4.27E-06 |  |
| rs4292285 | 4 | 145271954 | A | T | 0.399 | -0.129 | 0.047 | 5.82E-03 | -0.099 | 0.028 | 3.28E-04 |  |
| rs13139571 | 4 | 156645513 | A | C | 0.239 | -0.296 | 0.054 | 3.70E-08 | -0.231 | 0.032 | 3.06E-13 |  |
| rs869396 | 4 | 169688000 | A | C | 0.467 | -0.180 | 0.047 | 1.09E-04 | -0.008 | 0.027 | 7.75E-01 |  |
| rs2291435 | 4 | 38387395 | T | C | 0.525 | -0.242 | 0.046 | 1.74E-07 | -0.135 | 0.027 | 7.33E-07 |  |
| rs871606 | 4 | 54799245 | T | C | 0.894 | 0.360 | 0.076 | 2.22E-06 | -0.090 | 0.045 | 4.53E-02 |  |
| rs1458038 | 4 | 81164723 | T | C | 0.297 | 0.661 | 0.051 | 6.08E-39 | 0.411 | 0.030 | 3.48E-43 |  |
| rs2014912 | 4 | 86715670 | T | C | 0.152 | 0.512 | 0.064 | 1.80E-15 | 0.149 | 0.038 | 8.13E-05 |  |
| rs10077885 | 5 | 114390121 | A | C | 0.498 | -0.247 | 0.048 | 3.54E-07 | -0.132 | 0.029 | 3.55E-06 |  |
| rs13359291 | 5 | 122476457 | A | G | 0.165 | 0.401 | 0.062 | 1.06E-10 | 0.161 | 0.037 | 1.02E-05 |  |
| rs6891344 | 5 | 123136656 | A | G | 0.814 | 0.284 | 0.060 | 2.15E-06 | 0.218 | 0.035 | 7.19E-10 |  |
| rs6595838 | 5 | 127868199 | A | G | 0.289 | 0.236 | 0.051 | 3.14E-06 | 0.122 | 0.030 | 4.36E-05 |  |
| rs12521868 | 5 | 131784393 | T | G | 0.416 | -0.123 | 0.048 | 9.75E-03 | -0.146 | 0.028 | 1.81E-07 |  |
| rs9687065 | 5 | 148391140 | A | G | 0.804 | 0.250 | 0.059 | 2.28E-05 | 0.179 | 0.035 | 2.74E-07 |  |
| rs11953630 | 5 | 157845402 | T | C | 0.369 | -0.446 | 0.050 | 5.15E-19 | -0.219 | 0.030 | 1.20E-13 |  |
| rs72812846 | 5 | 173377636 | A | T | 0.278 | -0.238 | 0.053 | 8.24E-06 | -0.170 | 0.031 | 6.02E-08 |  |
| rs1173771 | 5 | 32815028 | A | G | 0.398 | -0.523 | 0.047 | 6.04E-29 | -0.258 | 0.028 | 7.96E-21 |  |
| rs10078021 | 5 | 75038431 | T | G | 0.626 | -0.062 | 0.049 | 2.06E-01 | -0.122 | 0.029 | 2.33E-05 |  |
| rs10057188 | 5 | 77837789 | A | G | 0.452 | -0.194 | 0.048 | 4.37E-05 | -0.067 | 0.028 | 1.65E-02 |  |
| rs10059921 | 5 | 87514515 | T | G | 0.085 | -0.373 | 0.092 | 4.89E-05 | -0.219 | 0.054 | 5.26E-05 |  |
| rs9372498 | 6 | 118572486 | A | T | 0.085 | 0.360 | 0.083 | 1.40E-05 | 0.194 | 0.049 | 7.35E-05 |  |
| rs11154027 | 6 | 121781390 | T | C | 0.449 | -0.009 | 0.048 | 8.50E-01 | -0.066 | 0.028 | 1.90E-02 |  |
| rs13209747 | 6 | 127115454 | T | C | 0.447 | 0.412 | 0.047 | 9.75E-19 | 0.254 | 0.027 | 2.04E-20 |  |
| rs9349379 | 6 | 12903957 | A | G | 0.591 | 0.262 | 0.049 | 6.37E-08 | 0.073 | 0.029 | 1.07E-02 | Excluded in MR-PRESSO (CAD-SBP) |
| rs17080102 | 6 | 151004770 | C | G | 0.068 | -0.718 | 0.092 | 4.94E-15 | -0.506 | 0.054 | 6.11E-21 |  |
| rs9479200 | 6 | 152398505 | A | G | 0.877 | -0.099 | 0.071 | 1.65E-01 | 0.149 | 0.042 | 3.49E-04 |  |
| rs449789 | 6 | 159699125 | C | G | 0.135 | 0.169 | 0.068 | 1.29E-02 | -0.043 | 0.040 | 2.87E-01 |  |
| rs73030266 | 6 | 166179459 | A | T | 0.933 | 0.313 | 0.098 | 1.40E-03 | 0.274 | 0.057 | 1.68E-06 |  |
| rs1322639 | 6 | 169587103 | A | G | 0.771 | 0.089 | 0.056 | 1.11E-01 | -0.131 | 0.033 | 6.37E-05 |  |
| rs6911827 | 6 | 22130601 | T | C | 0.462 | 0.152 | 0.047 | 1.30E-03 | 0.120 | 0.028 | 1.66E-05 |  |
| rs1799945 | 6 | 26091179 | C | G | 0.852 | -0.543 | 0.066 | 1.32E-16 | -0.379 | 0.039 | 1.04E-22 |  |
| rs409558 | 6 | 31708147 | T | C | 0.829 | 0.446 | 0.064 | 2.63E-12 | 0.135 | 0.038 | 3.51E-04 |  |
| rs1563788 | 6 | 43308363 | T | C | 0.294 | 0.306 | 0.050 | 9.79E-10 | 0.130 | 0.030 | 1.05E-05 |  |
| rs78648104 | 6 | 50683009 | T | C | 0.899 | -0.357 | 0.083 | 1.69E-05 | -0.182 | 0.049 | 1.97E-04 |  |
| rs13205180 | 6 | 51832494 | T | C | 0.481 | 0.041 | 0.046 | 3.78E-01 | 0.120 | 0.027 | 1.25E-05 |  |
| rs1925153 | 6 | 56102780 | T | C | 0.440 | -0.051 | 0.049 | 2.99E-01 | 0.072 | 0.029 | 1.21E-02 |  |
| rs10943605 | 6 | 79655477 | A | G | 0.489 | 0.175 | 0.046 | 1.58E-04 | 0.155 | 0.027 | 1.16E-08 |  |
| rs35410524 | 6 | 96885405 | T | C | 0.192 | 0.300 | 0.059 | 3.38E-07 | 0.141 | 0.035 | 4.64E-05 |  |
| rs17477177 | 7 | 106411858 | T | C | 0.791 | -0.564 | 0.056 | 1.60E-23 | -0.015 | 0.033 | 6.45E-01 |  |
| rs4728142 | 7 | 128573967 | A | G | 0.438 | -0.216 | 0.047 | 3.91E-06 | -0.075 | 0.028 | 6.18E-03 |  |
| rs11556924 | 7 | 129663496 | T | C | 0.371 | -0.249 | 0.050 | 5.92E-07 | -0.142 | 0.029 | 1.31E-06 | Excluded in MR-PRESSO (CAD-SBP) |
| rs13238550 | 7 | 131059056 | A | G | 0.391 | 0.170 | 0.047 | 3.29E-04 | 0.089 | 0.028 | 1.29E-03 |  |
| rs7810028 | 7 | 139461616 | C | G | 0.806 | 0.221 | 0.058 | 1.47E-04 | 0.147 | 0.034 | 1.78E-05 |  |
| rs3918226 | 7 | 150690176 | T | C | 0.081 | 0.547 | 0.091 | 1.84E-09 | 0.545 | 0.053 | 1.62E-24 | Excluded in MR-PRESSO (CAD-SBP) |
| rs891511 | 7 | 150704843 | A | G | 0.352 | -0.253 | 0.052 | 1.23E-06 | -0.211 | 0.031 | 5.54E-12 |  |
| rs10224002 | 7 | 151415041 | A | G | 0.719 | -0.238 | 0.053 | 5.99E-06 | -0.137 | 0.031 | 1.01E-05 |  |
| rs2107595 | 7 | 19049388 | A | G | 0.166 | 0.302 | 0.062 | 1.24E-06 | -0.052 | 0.037 | 1.57E-01 | Excluded in MR-PRESSO (CAD-SBP) |
| rs2969070 | 7 | 2512545 | A | G | 0.637 | -0.275 | 0.048 | 8.38E-09 | -0.198 | 0.028 | 1.85E-12 |  |
| rs1055144 | 7 | 25871109 | T | C | 0.193 | 0.108 | 0.058 | 6.34E-02 | 0.000 | 0.034 | 9.96E-01 |  |
| rs6969780 | 7 | 27159136 | C | G | 0.096 | 0.370 | 0.079 | 3.12E-06 | 0.203 | 0.047 | 1.28E-05 |  |
| rs7777128 | 7 | 27337113 | C | G | 0.082 | 0.550 | 0.084 | 4.67E-11 | 0.331 | 0.049 | 1.74E-11 |  |
| rs917275 | 7 | 28658522 | A | G | 0.611 | -0.113 | 0.048 | 1.79E-02 | 0.035 | 0.028 | 2.13E-01 |  |
| rs76206723 | 7 | 40447971 | A | G | 0.110 | -0.336 | 0.074 | 6.44E-06 | -0.033 | 0.044 | 4.54E-01 |  |
| rs11977526 | 7 | 46008110 | A | G | 0.399 | -0.254 | 0.048 | 1.64E-07 | 0.051 | 0.029 | 7.28E-02 | Excluded in MR-PRESSO (CAD-SBP) |
| rs2282978 | 7 | 92264410 | T | C | 0.658 | 0.070 | 0.049 | 1.48E-01 | -0.052 | 0.029 | 7.17E-02 |  |
| rs2978098 | 8 | 101676675 | A | C | 0.558 | 0.148 | 0.047 | 1.73E-03 | 0.122 | 0.028 | 1.16E-05 |  |
| rs35783704 | 8 | 105966258 | A | G | 0.109 | -0.522 | 0.077 | 1.50E-11 | -0.241 | 0.045 | 1.08E-07 |  |
| rs2898290 | 8 | 11433909 | T | C | 0.484 | 0.342 | 0.047 | 2.12E-13 | 0.132 | 0.027 | 1.33E-06 |  |
| rs2071518 | 8 | 120435812 | T | C | 0.257 | 0.218 | 0.052 | 3.13E-05 | -0.141 | 0.031 | 4.68E-06 |  |
| rs894344 | 8 | 135612745 | A | G | 0.590 | -0.146 | 0.047 | 1.76E-03 | -0.092 | 0.027 | 8.27E-04 |  |
| rs4454254 | 8 | 141060027 | A | G | 0.635 | -0.221 | 0.048 | 3.24E-06 | -0.019 | 0.028 | 5.07E-01 |  |
| rs34591516 | 8 | 142367087 | T | C | 0.053 | 0.561 | 0.107 | 1.48E-07 | 0.287 | 0.063 | 5.69E-06 |  |
| rs62524579 | 8 | 144060955 | A | G | 0.531 | -0.181 | 0.053 | 6.86E-04 | -0.150 | 0.031 | 1.14E-06 |  |
| rs6557876 | 8 | 25900675 | T | C | 0.251 | -0.367 | 0.053 | 5.98E-12 | -0.197 | 0.031 | 3.60E-10 |  |
| rs2978456 | 8 | 42324765 | T | C | 0.556 | -0.107 | 0.050 | 3.17E-02 | -0.012 | 0.029 | 6.78E-01 |  |
| rs1449544 | 8 | 76591880 | A | C | 0.542 | 0.222 | 0.046 | 1.40E-06 | 0.037 | 0.027 | 1.74E-01 |  |
| rs111245230 | 9 | 113169775 | T | C | 0.966 | -0.692 | 0.130 | 9.99E-08 | -0.400 | 0.078 | 2.61E-07 |  |
| rs1953126 | 9 | 123640500 | T | C | 0.355 | 0.184 | 0.048 | 1.22E-04 | 0.014 | 0.029 | 6.23E-01 |  |
| rs10818775 | 9 | 125755571 | T | C | 0.124 | -0.273 | 0.070 | 8.68E-05 | -0.026 | 0.042 | 5.38E-01 |  |
| rs72765298 | 9 | 127900996 | T | C | 0.882 | -0.348 | 0.073 | 1.65E-06 | -0.041 | 0.043 | 3.39E-01 |  |
| rs687621 | 9 | 136137065 | A | G | 0.661 | 0.098 | 0.048 | 4.29E-02 | 0.133 | 0.029 | 4.87E-06 |  |
| rs6271 | 9 | 136522274 | T | C | 0.073 | -0.434 | 0.102 | 2.11E-05 | -0.365 | 0.061 | 2.72E-09 |  |
| rs4364717 | 9 | 21801530 | A | G | 0.547 | -0.107 | 0.046 | 1.92E-02 | -0.086 | 0.027 | 1.67E-03 | Excluded in MR-PRESSO (CAD-SBP) |
| rs76452347 | 9 | 35906471 | T | C | 0.206 | -0.172 | 0.062 | 5.85E-03 | -0.169 | 0.037 | 5.51E-06 |  |

**Table S3.** The genetic variants derived from non-UK Biobank studies without adjustment for body mass index that were used as instruments for systolic blood pressure in sensitivity analyses.

| RSID | Chromosome | Position | Effect allele | Other allele | Beta (mmHg) | Standard error | P |
| --- | --- | --- | --- | --- | --- | --- | --- |
| rs11105354 | 12 | 90026523 | A | G | 0.8339 | 0.0978 | 1.49E-17 |
| rs1173770 | 5 | 32821211 | T | C | -0.6129 | 0.0793 | 1.10E-14 |
| rs167479 | 19 | 11526765 | T | G | -0.6470 | 0.0838 | 1.19E-14 |
| rs17367504 | 1 | 11862778 | A | G | 0.7601 | 0.0992 | 1.87E-14 |
| rs2392929 | 7 | 106414069 | T | G | -0.6347 | 0.0896 | 1.44E-12 |
| rs34328549 | 19 | 7253184 | A | G | -0.8409 | 0.1241 | 1.25E-11 |
| rs1216743 | 11 | 100573120 | A | G | 0.5408 | 0.0802 | 1.57E-11 |
| rs3781956 | 11 | 1945884 | A | G | 0.5051 | 0.0779 | 8.69E-11 |
| rs10857147 | 4 | 81181072 | A | T | -0.5333 | 0.0852 | 3.96E-10 |
| rs111267152 | 20 | 57704603 | A | G | 0.6983 | 0.1130 | 6.43E-10 |
| rs2071265 | 7 | 27238691 | C | G | -0.8319 | 0.1351 | 7.47E-10 |
| rs62434125 | 6 | 151002371 | T | C | 0.8541 | 0.1423 | 1.97E-09 |
| rs1731249 | 2 | 26920025 | A | T | -0.7053 | 0.1199 | 4.02E-09 |
| rs7497304 | 15 | 91429176 | T | G | 0.5110 | 0.0869 | 4.05E-09 |
| rs6941056 | 6 | 143591821 | C | G | 0.4287 | 0.0730 | 4.36E-09 |
| rs17010961 | 4 | 86723103 | A | T | 0.5902 | 0.1008 | 4.72E-09 |
| rs9508499 | 13 | 30162353 | A | G | -0.5031 | 0.0903 | 2.55E-08 |
| rs1834013 | 16 | 75496163 | T | G | 0.4381 | 0.0791 | 3.09E-08 |
| rs2416971 | 9 | 128466113 | A | G | 0.4148 | 0.0754 | 3.71E-08 |
| rs448798 | 2 | 165052533 | A | G | -0.4039 | 0.0734 | 3.78E-08 |
| rs57301765 | 7 | 19052733 | A | G | 0.5383 | 0.0985 | 4.64E-08 |

**Table S4.** The genetic variants derived from non-UK Biobank studies without adjustment for body mass index that were used as instruments for diastolic blood pressure in sensitivity analyses.

| RSID | Chromosome | Position | Effect allele | Other allele | Beta (mmHg) | Standard error | P |
| --- | --- | --- | --- | --- | --- | --- | --- |
| rs6015450 | 20 | 57751117 | A | G | -0.5502 | 0.0740 | 1.07E-13 |
| rs167479 | 19 | 11526765 | T | G | -0.4206 | 0.0572 | 1.86E-13 |
| rs16998073 | 4 | 81184341 | A | T | -0.4058 | 0.0582 | 3.26E-12 |
| rs597808 | 12 | 111973358 | A | G | 0.3749 | 0.0542 | 4.61E-12 |
| rs3980310 | 10 | 95982015 | A | G | 0.3684 | 0.0538 | 7.82E-12 |
| rs2193950 | 5 | 32820553 | T | C | -0.3706 | 0.0551 | 1.67E-11 |
| rs3918226 | 7 | 150690176 | T | C | 0.6219 | 0.0953 | 6.62E-11 |
| rs45603435 | 1 | 11861855 | A | G | 0.4554 | 0.0699 | 7.37E-11 |
| rs67257108 | 11 | 10157776 | A | G | -0.4311 | 0.0664 | 8.50E-11 |
| rs62434129 | 6 | 151008837 | A | T | 0.6350 | 0.0991 | 1.48E-10 |
| rs1528293 | 3 | 169154511 | A | T | 0.3350 | 0.0526 | 1.95E-10 |
| rs11513729 | 12 | 112273499 | T | C | 0.3531 | 0.0555 | 2.02E-10 |
| rs62145083 | 2 | 43068738 | A | C | -0.5522 | 0.0879 | 3.35E-10 |
| rs11066309 | 12 | 112883476 | A | G | 0.3404 | 0.0558 | 1.09E-09 |
| rs11066188 | 12 | 112610714 | A | G | 0.3331 | 0.0548 | 1.22E-09 |
| rs7497304 | 15 | 91429176 | T | G | 0.3567 | 0.0597 | 2.25E-09 |
| rs12258967 | 10 | 18727959 | C | G | 0.3433 | 0.0584 | 4.24E-09 |
| rs1888693 | 10 | 18440444 | A | G | 0.3138 | 0.0536 | 4.75E-09 |
| rs12978472 | 19 | 7257990 | C | G | 0.4766 | 0.0818 | 5.75E-09 |
| rs7674212 | 4 | 103988899 | T | G | -0.3033 | 0.0522 | 6.10E-09 |
| rs77149258 | 16 | 88590013 | C | G | 0.9932 | 0.1749 | 1.37E-08 |
| rs79217743 | 15 | 75117912 | T | G | 0.4057 | 0.0718 | 1.57E-08 |
| rs1731249 | 2 | 26920025 | A | T | -0.3110 | 0.0556 | 2.17E-08 |
| rs1316379 | 5 | 157889391 | A | G | 0.3185 | 0.0569 | 2.20E-08 |
| rs223490 | 4 | 103679117 | T | G | -0.2995 | 0.0536 | 2.30E-08 |
| rs12940887 | 17 | 47402807 | T | C | 0.3071 | 0.0559 | 3.92E-08 |
| rs7136259 | 12 | 90081188 | T | C | -0.2854 | 0.0520 | 4.00E-08 |

**Table S5**. Distribution of risk factors for individuals in the top and bottom deciles of residual systolic blood pressure (SBP) and diastolic blood pressure (DBP) that had a weighted allele score above and below the population median in the main analysis. BMI: body mass index; LDL-C: low-density lipoprotein cholesterol; SD: standard deviation.

|  | **Top decile** | | | | | | **Bottom decile** | | | | | |
| --- | --- | --- | --- | --- | --- | --- | --- | --- | --- | --- | --- | --- |
|  | **SBP weighted allele score** | | | **DBP weighted allele score** | | | **SBP weighted allele score** | | | **DBP weighted allele score** | | |
|  | **Below median** | **Above median** | **P** | **Below median** | **Above median** | **P** | **Below median** | **Above median** | **P** | **Below median** | **Above median** | **P** |
| **Age, mean (SD), y** | 57.6 (7.7) | 56.9 (7.8) | <0.001 | 56.0 (7.6) | 55.3 (7.7) | <0.001 | 58.4 (7.1) | 58.2 (7.2) | <0.001 | 56.1 (8.4) | 55.8 (8.4) | 0.007 |
| **Sex, N (%)** |  |  | 0.006 |  |  | 0.09 |  |  | 0.95 |  |  | 0.76 |
| **Male** | 5514 (40.7) | 5293 (39.1) |  | 5790 (43.0) | 5769 (42.9) |  | 5178 (40.6) | 5311 (41.6) |  | 5582 (43.5) | 5607 (43.8) |  |
| **Female** | 8011 (59.3) | 8232 (60.9) |  | 7655 (57.0) | 7676 (57.1) |  | 7583 (59.4) | 7450 (58.4) |  | 7233 (56.5) | 7208 (56.2) |  |
| **Socioeconomic status, N (%)*** |  |  | 0.49 |  |  | 0.81 |  |  | 0.33 |  |  | 0.64 |
| **Quintile 1** | 2926 (21.6) | 2916 (21.6) |  | 2721 (20.2) | 2810 (20.9) |  | 2777 (21.8) | 2819 (22.1) |  | 2800 (21.8) | 2849 (22.2) |  |
| **Quintile 2-4** | 8379 (61.9) | 8457 (62.5) |  | 8325 (61.9) | 8296 (61.7) |  | 7723 (60.5) | 7683 (60.2) |  | 7782 (60.7) | 7778 (60.7) |  |
| **Quintile 5** | 2220 (16.4) | 2152 (15.9) |  | 2399 (17.8) | 2339 (17.4) |  | 2261 (17.7) | 2259 (17.7) |  | 2233 (17.4) | 2188 (17.1) |  |
| **Smoking index, mean (SD)** | 0.4 (0.7) | 0.4 (0.7) | 0.37 | 0.4 (0.7) | 0.4 (0.7) | 0.10 | 0.4 (0.8) | 0.4 (0.8) | 0.21 | 0.4 (0.7) | 0.4 (0.7) | 0.14 |
| **BMI, mean (SD), kg/m^2^** | 28.3 (5.0) | 27.9 (4.8) | <0.001 | 29.5 (5.3) | 28.9 (5.1) | 0.07 | 25.0 (3.7) | 24.9 (3.8) | <0.001 | 24.5 (3.4) | 24.4 (3.4) | 0.59 |
| **SBP, mean (SD), mmHg** | 168.8 (12.0) | 172.8 (12.5) | <0.001 | 159.3 (16.1) | 162.7 (16.7) | <0.001 | 110.4 (7.5) | 113.4 (7.9) | <0.001 | 116.6 (13.0) | 119.0 (13.3) | <0.001 |
| **DBP, mean (SD), mmHg** | 94.1 (9.3) | 95.7 (9.7) | <0.001 | 98.9 (5.5) | 101.3 (5.7) | <0.001 | 69.2 (6.4) | 70.8 (6.6) | <0.001 | 64.8 (3.9) | 66.8 (4.0) | <0.001 |
| **Diabetes diagnosed, N (%)** | 282 (2.1) | 226 (1.7) | 0.01 | 255 (1.9) | 183 (1.4) | 0.04 | 222 (1.7) | 268 (2.1) | <0.001 | 330 (2.6) | 344 (2.7) | 0.61 |
| **LDL-C, mean (SD), mmol/L** | 3.9 (0.9) | 3.9 (0.9) | 0.06 | 3.9 (0.8) | 3.9 (0.9) | 0.09 | 3.5 (0.8) | 3.5 (0.8) | 0.14 | 3.4 (0.8) | 3.4 (0.8) | 0.77 |

*Socioeconomic status quintiles according to Townsend deprivation index combining information on social class, employment, car availability and housing.

**Table S6.** Results of linear Mendelian randomization (MR) sensitivity analyses investigating associations of genetically proxied blood pressure with risk of cardiovascular disease outcomes. IVW: random effects inverse-variance weighted model.

| **Exposure** | **Method** | **Cardiovascular disease** | | | **Coronary artery disease** | | | **Stroke** | | |
| --- | --- | --- | --- | --- | --- | --- | --- | --- | --- | --- |
|  |  | **Hazard ratio** | **95% confidence interval** | **P** | **Hazard ratio** | **95% confidence interval** | **P** | **Hazard ratio** | **95% confidence interval** | **P** |
| Systolic blood pressure (per 10mmHg increase) | Allele score | 1.49 | 1.38-1.61 | 6.58E-25 | 1.50 | 1.38-1.63 | 2.33E-21 | 1.44 | 1.22-1.70 | 1.24E-05 |
|  | IVW | 1.51 | 1.40-1.63 | 5.30E-25 | 1.48 | 1.34-1.63 | 7.00E-26 | 1.55 | 1.31-1.82 | 1.70E-07 |
|  | Weighted median | 1.50 | 1.39-1.63 | 4.36E-25 | 1.49 | 1.38-1.62 | 2.74E-22 | 1.58 | 1.29-1.93 | 2.03E-07 |
|  | MR-PRESSO | 1.56 | 1.46-1.67 | 8.44E-30 | 1.59 | 1.48-1.71 | 1.70E-28 | 1.43 | 1.27-1.60 | 3.21E-09 |
|  | MR-Egger intercept |  | | 0.49 |  | | 0.31 |  | | 0.22 |
|  | MR-Egger | 1.54 | 1.30-1.82 | 7.90E-07 | 1.51 | 1.26-1.81 | 1.17E-05 | 1.64 | 1.30-2.04 | 2.89E-05 |
| Diastolic blood pressure (per 5mmHg increase) | Allele score | 1.35 | 1.29-1.42 | 5.48E-34 | 1.36 | 1.26-1.47 | 1.26E-15 | 1.39 | 1.20-1.62 | 1.74E-05 |
|  | IVW | 1.35 | 1.26-1.44 | 3.70E-18 | 1.39 | 1.30-1.50 | 3.50E-20 | 1.40 | 1.22-1.61 | 1.60E-06 |
|  | Weighted median | 1.43 | 1.32-1.53 | 3.78E-19 | 1.43 | 1.32-1.56 | 1.33E-18 | 1.43 | 1.30-1.50 | 4.44E-06 |
|  | MR-PRESSO | 1.40 | 1.32-1.48 | 6.69E-24 | 1.41 | 1.32-1.50 | 3.32E-21 | 1.33 | 1.21-1.46 | 1.13E-08 |
|  | MR-Egger intercept |  | | 0.06 |  | | 0.43 |  | | 0.19 |
|  | MR-Egger | 1.65 | 1.44-1.87 | 1.98E-12 | 1.68 | 1.46-1.94 | 1.27E-12 | 1.46 | 1.23-1.74 | 1.69E-05 |

**Table S7.** Results of non-linear Mendelian randomization analyses investigating associations of genetically proxied systolic blood pressure with risk of cardiovascular disease outcomes. Hazard ratios are given per 1mmHg increase in genetically proxied blood pressure.

| **Centile** | **Cardiovascular disease** | | | **Coronary artery disease** | | | **Stroke** | | |
| --- | --- | --- | --- | --- | --- | --- | --- | --- | --- |
|  | **Hazard ratio** | **Lower 95% confidence interval** | **Upper 95% confidence interval** | **Hazard ratio** | **Lower 95% confidence interval** | **Upper 95% confidence interval** | **Hazard ratio** | **Lower 95% confidence interval** | **Upper 95% confidence interval** |
| 1 | 1.05 | 1.00 | 1.10 | 1.08 | 1.00 | 1.17 | 0.97 | 0.85 | 1.09 |
| 2 | 1.02 | 0.97 | 1.08 | 1.07 | 0.98 | 1.16 | 0.94 | 0.79 | 1.11 |
| 3 | 1.08 | 1.03 | 1.14 | 1.09 | 1.01 | 1.18 | 1.10 | 0.93 | 1.31 |
| 4 | 1.01 | 0.96 | 1.07 | 1.03 | 0.94 | 1.13 | 1.01 | 0.87 | 1.19 |
| 5 | 1.05 | 1.00 | 1.11 | 1.05 | 0.96 | 1.15 | 1.08 | 0.89 | 1.31 |
| 6 | 1.05 | 0.99 | 1.12 | 1.09 | 0.99 | 1.20 | 0.90 | 0.75 | 1.09 |
| 7 | 1.07 | 1.01 | 1.13 | 1.09 | 0.99 | 1.19 | 1.05 | 0.88 | 1.26 |
| 8 | 0.99 | 0.93 | 1.05 | 1.00 | 0.91 | 1.10 | 0.96 | 0.78 | 1.19 |
| 9 | 1.04 | 0.98 | 1.10 | 1.06 | 0.97 | 1.17 | 0.98 | 0.78 | 1.23 |
| 10 | 1.06 | 1.00 | 1.12 | 1.02 | 0.93 | 1.12 | 1.17 | 0.94 | 1.47 |
| 11 | 1.06 | 1.00 | 1.13 | 1.02 | 0.94 | 1.12 | 1.26 | 1.04 | 1.53 |
| 12 | 1.00 | 0.94 | 1.06 | 0.97 | 0.88 | 1.06 | 1.12 | 0.93 | 1.35 |
| 13 | 1.09 | 1.02 | 1.16 | 1.11 | 1.00 | 1.23 | 1.15 | 0.96 | 1.38 |
| 14 | 1.04 | 0.98 | 1.10 | 1.01 | 0.92 | 1.10 | 1.13 | 0.93 | 1.38 |
| 15 | 1.04 | 0.98 | 1.10 | 1.03 | 0.94 | 1.13 | 1.12 | 0.97 | 1.29 |
| 16 | 1.04 | 0.97 | 1.11 | 1.10 | 1.00 | 1.22 | 0.82 | 0.67 | 1.00 |
| 17 | 1.03 | 0.97 | 1.09 | 1.03 | 0.93 | 1.14 | 1.11 | 0.92 | 1.34 |
| 18 | 1.02 | 0.96 | 1.08 | 1.02 | 0.93 | 1.13 | 1.02 | 0.86 | 1.21 |
| 19 | 0.96 | 0.90 | 1.02 | 0.95 | 0.86 | 1.05 | 0.95 | 0.79 | 1.15 |
| 20 | 1.12 | 1.06 | 1.19 | 1.17 | 1.07 | 1.28 | 0.99 | 0.82 | 1.19 |
| 21 | 0.97 | 0.91 | 1.03 | 0.99 | 0.90 | 1.09 | 0.90 | 0.74 | 1.09 |
| 22 | 1.00 | 0.94 | 1.06 | 0.97 | 0.88 | 1.07 | 1.10 | 0.92 | 1.31 |
| 23 | 0.99 | 0.93 | 1.05 | 1.00 | 0.91 | 1.10 | 0.92 | 0.78 | 1.09 |
| 24 | 1.04 | 0.98 | 1.11 | 1.06 | 0.97 | 1.17 | 0.95 | 0.77 | 1.17 |
| 25 | 1.05 | 0.98 | 1.12 | 1.02 | 0.93 | 1.12 | 1.16 | 0.94 | 1.44 |
| 26 | 1.18 | 1.12 | 1.26 | 1.21 | 1.10 | 1.33 | 1.08 | 0.91 | 1.27 |
| 27 | 1.02 | 0.95 | 1.09 | 1.06 | 0.95 | 1.17 | 0.93 | 0.75 | 1.15 |
| 28 | 0.99 | 0.93 | 1.05 | 0.99 | 0.91 | 1.08 | 0.87 | 0.72 | 1.05 |
| 29 | 1.02 | 0.96 | 1.08 | 1.01 | 0.92 | 1.11 | 1.11 | 0.87 | 1.42 |
| 30 | 1.07 | 1.01 | 1.13 | 1.10 | 1.01 | 1.20 | 0.92 | 0.78 | 1.10 |
| 31 | 1.03 | 0.97 | 1.09 | 0.98 | 0.90 | 1.06 | 1.35 | 1.12 | 1.62 |
| 32 | 1.07 | 1.01 | 1.13 | 1.10 | 1.01 | 1.20 | 1.00 | 0.85 | 1.18 |
| 33 | 1.03 | 0.98 | 1.09 | 1.04 | 0.96 | 1.13 | 1.02 | 0.86 | 1.22 |
| 34 | 1.01 | 0.95 | 1.08 | 1.05 | 0.96 | 1.15 | 0.80 | 0.61 | 1.04 |
| 35 | 1.08 | 1.02 | 1.14 | 1.11 | 1.02 | 1.22 | 0.99 | 0.79 | 1.24 |
| 36 | 1.08 | 1.02 | 1.14 | 1.10 | 1.00 | 1.20 | 1.08 | 0.91 | 1.27 |
| 37 | 1.04 | 0.98 | 1.11 | 1.05 | 0.96 | 1.16 | 1.08 | 0.87 | 1.34 |
| 38 | 1.03 | 0.97 | 1.09 | 1.00 | 0.92 | 1.10 | 1.18 | 0.96 | 1.46 |
| 39 | 1.02 | 0.96 | 1.07 | 1.03 | 0.94 | 1.12 | 1.02 | 0.86 | 1.21 |
| 40 | 1.04 | 0.98 | 1.10 | 1.08 | 0.99 | 1.19 | 0.90 | 0.77 | 1.06 |
| 41 | 1.00 | 0.95 | 1.07 | 0.99 | 0.91 | 1.08 | 1.31 | 1.04 | 1.65 |
| 42 | 1.02 | 0.96 | 1.09 | 1.04 | 0.95 | 1.14 | 0.91 | 0.73 | 1.13 |
| 43 | 0.96 | 0.90 | 1.02 | 0.95 | 0.86 | 1.05 | 0.98 | 0.76 | 1.27 |
| 44 | 1.02 | 0.96 | 1.09 | 1.03 | 0.94 | 1.14 | 1.02 | 0.85 | 1.22 |
| 45 | 1.07 | 1.01 | 1.13 | 1.06 | 0.97 | 1.16 | 1.01 | 0.82 | 1.25 |
| 46 | 1.01 | 0.95 | 1.08 | 1.01 | 0.92 | 1.12 | 0.99 | 0.83 | 1.18 |
| 47 | 1.12 | 1.05 | 1.19 | 1.18 | 1.06 | 1.30 | 1.02 | 0.85 | 1.22 |
| 48 | 1.06 | 1.00 | 1.11 | 1.06 | 0.98 | 1.15 | 1.04 | 0.85 | 1.28 |
| 49 | 1.02 | 0.96 | 1.08 | 1.06 | 0.96 | 1.15 | 0.91 | 0.77 | 1.08 |
| 50 | 1.07 | 1.01 | 1.13 | 1.05 | 0.96 | 1.15 | 1.06 | 0.89 | 1.27 |
| 51 | 1.03 | 0.97 | 1.10 | 1.03 | 0.93 | 1.14 | 0.98 | 0.81 | 1.19 |
| 52 | 1.11 | 1.05 | 1.18 | 1.11 | 1.01 | 1.22 | 1.07 | 0.90 | 1.28 |
| 53 | 1.06 | 1.00 | 1.12 | 1.09 | 1.00 | 1.19 | 0.99 | 0.84 | 1.17 |
| 54 | 1.07 | 1.01 | 1.14 | 1.07 | 0.97 | 1.18 | 1.26 | 1.04 | 1.52 |
| 55 | 1.01 | 0.95 | 1.07 | 0.99 | 0.90 | 1.09 | 1.10 | 0.91 | 1.32 |
| 56 | 0.99 | 0.94 | 1.04 | 0.97 | 0.89 | 1.06 | 1.11 | 0.92 | 1.36 |
| 57 | 1.02 | 0.97 | 1.07 | 1.02 | 0.95 | 1.11 | 1.02 | 0.85 | 1.22 |
| 58 | 1.00 | 0.95 | 1.06 | 0.99 | 0.90 | 1.09 | 0.98 | 0.84 | 1.15 |
| 59 | 1.01 | 0.96 | 1.07 | 1.03 | 0.94 | 1.13 | 0.90 | 0.74 | 1.11 |
| 60 | 1.03 | 0.97 | 1.09 | 1.08 | 0.98 | 1.18 | 0.82 | 0.67 | 1.00 |
| 61 | 0.96 | 0.91 | 1.02 | 0.95 | 0.87 | 1.04 | 1.07 | 0.90 | 1.27 |
| 62 | 1.00 | 0.95 | 1.06 | 0.99 | 0.91 | 1.07 | 0.98 | 0.82 | 1.17 |
| 63 | 0.98 | 0.93 | 1.03 | 0.98 | 0.90 | 1.07 | 1.00 | 0.85 | 1.19 |
| 64 | 1.03 | 0.98 | 1.08 | 1.05 | 0.97 | 1.14 | 0.96 | 0.81 | 1.14 |
| 65 | 1.04 | 0.99 | 1.09 | 1.05 | 0.97 | 1.14 | 1.03 | 0.87 | 1.22 |
| 66 | 0.98 | 0.93 | 1.03 | 0.97 | 0.89 | 1.05 | 1.01 | 0.87 | 1.17 |
| 67 | 1.08 | 1.03 | 1.13 | 1.04 | 0.97 | 1.12 | 1.32 | 1.13 | 1.55 |
| 68 | 1.05 | 1.00 | 1.11 | 1.04 | 0.96 | 1.14 | 1.09 | 0.94 | 1.27 |
| 69 | 1.01 | 0.96 | 1.06 | 1.03 | 0.95 | 1.12 | 0.99 | 0.82 | 1.20 |
| 70 | 0.98 | 0.93 | 1.04 | 0.97 | 0.89 | 1.06 | 1.02 | 0.86 | 1.19 |
| 71 | 1.06 | 1.01 | 1.12 | 1.07 | 0.99 | 1.16 | 1.07 | 0.89 | 1.27 |
| 72 | 1.07 | 1.01 | 1.13 | 1.10 | 1.01 | 1.20 | 0.97 | 0.80 | 1.18 |
| 73 | 1.08 | 1.03 | 1.14 | 1.06 | 0.98 | 1.14 | 1.22 | 1.04 | 1.42 |
| 74 | 1.07 | 1.02 | 1.12 | 1.08 | 1.01 | 1.17 | 1.00 | 0.85 | 1.18 |
| 75 | 1.06 | 1.01 | 1.12 | 1.04 | 0.96 | 1.12 | 1.18 | 0.99 | 1.39 |
| 76 | 1.11 | 1.05 | 1.16 | 1.08 | 1.00 | 1.17 | 1.15 | 0.98 | 1.35 |
| 77 | 1.06 | 1.00 | 1.13 | 1.08 | 0.98 | 1.18 | 0.98 | 0.83 | 1.16 |
| 78 | 1.03 | 0.98 | 1.08 | 1.02 | 0.94 | 1.11 | 1.08 | 0.93 | 1.24 |
| 79 | 1.04 | 0.99 | 1.09 | 1.02 | 0.95 | 1.10 | 1.07 | 0.91 | 1.27 |
| 80 | 1.03 | 0.98 | 1.08 | 1.03 | 0.96 | 1.11 | 1.00 | 0.86 | 1.16 |
| 81 | 1.09 | 1.03 | 1.14 | 1.09 | 1.00 | 1.18 | 1.01 | 0.87 | 1.17 |
| 82 | 1.03 | 0.98 | 1.08 | 1.05 | 0.97 | 1.14 | 1.02 | 0.89 | 1.17 |
| 83 | 1.06 | 1.01 | 1.12 | 1.06 | 0.98 | 1.15 | 1.04 | 0.90 | 1.21 |
| 84 | 1.02 | 0.97 | 1.07 | 1.00 | 0.92 | 1.07 | 1.06 | 0.92 | 1.22 |
| 85 | 1.05 | 1.00 | 1.10 | 1.04 | 0.96 | 1.12 | 1.09 | 0.94 | 1.26 |
| 86 | 1.07 | 1.02 | 1.12 | 1.08 | 1.01 | 1.16 | 1.04 | 0.89 | 1.21 |
| 87 | 1.08 | 1.02 | 1.14 | 1.05 | 0.96 | 1.14 | 1.12 | 0.98 | 1.28 |
| 88 | 1.07 | 1.02 | 1.12 | 1.06 | 0.98 | 1.14 | 1.07 | 0.93 | 1.22 |
| 89 | 1.04 | 0.99 | 1.08 | 1.04 | 0.97 | 1.11 | 1.01 | 0.87 | 1.17 |
| 90 | 1.00 | 0.96 | 1.05 | 0.99 | 0.92 | 1.07 | 1.02 | 0.88 | 1.19 |
| 91 | 1.02 | 0.97 | 1.07 | 1.02 | 0.95 | 1.11 | 1.01 | 0.87 | 1.18 |
| 92 | 1.03 | 0.98 | 1.07 | 1.00 | 0.93 | 1.08 | 1.06 | 0.92 | 1.22 |
| 93 | 1.08 | 1.04 | 1.13 | 1.08 | 1.01 | 1.16 | 0.92 | 0.79 | 1.07 |
| 94 | 1.05 | 1.00 | 1.10 | 1.06 | 0.99 | 1.15 | 0.98 | 0.84 | 1.14 |
| 95 | 1.00 | 0.95 | 1.05 | 0.99 | 0.92 | 1.07 | 1.01 | 0.86 | 1.18 |
| 96 | 1.06 | 1.02 | 1.11 | 1.06 | 0.99 | 1.13 | 1.07 | 0.95 | 1.21 |
| 97 | 1.08 | 1.03 | 1.12 | 1.07 | 1.00 | 1.15 | 1.06 | 0.93 | 1.22 |
| 98 | 1.00 | 0.95 | 1.04 | 1.01 | 0.94 | 1.09 | 1.00 | 0.89 | 1.14 |
| 99 | 1.01 | 0.96 | 1.06 | 0.98 | 0.91 | 1.06 | 1.14 | 1.00 | 1.29 |
| 100 | 1.06 | 1.02 | 1.10 | 1.05 | 0.99 | 1.12 | 1.06 | 0.95 | 1.18 |

**Table S8.** Results of non-linear Mendelian randomization analyses investigating associations of genetically proxied diastolic blood pressure with risk of cardiovascular disease outcomes. Hazard ratios are given per 1mmHg increase in genetically proxied blood pressure.

| **Centile** | **Cardiovascular disease** | | | **Coronary artery disease** | | | **Stroke** | | |
| --- | --- | --- | --- | --- | --- | --- | --- | --- | --- |
|  | **Hazard ratio** | **Lower 95% confidence interval** | **Upper 95% confidence interval** | **Hazard ratio** | **Lower 95% confidence interval** | **Upper 95% confidence interval** | **Hazard ratio** | **Lower 95% confidence interval** | **Upper 95% confidence interval** |
| 1 | 1.12 | 1.02 | 1.23 | 1.18 | 1.03 | 1.36 | 1.26 | 0.92 | 1.73 |
| 2 | 1.07 | 0.98 | 1.18 | 1.05 | 0.90 | 1.22 | 1.12 | 0.87 | 1.45 |
| 3 | 1.10 | 0.99 | 1.22 | 1.13 | 0.96 | 1.34 | 0.97 | 0.71 | 1.34 |
| 4 | 1.14 | 1.03 | 1.26 | 1.17 | 1.00 | 1.37 | 0.98 | 0.72 | 1.32 |
| 5 | 1.11 | 1.00 | 1.22 | 1.15 | 0.98 | 1.35 | 1.16 | 0.88 | 1.54 |
| 6 | 1.09 | 0.98 | 1.21 | 1.18 | 1.00 | 1.41 | 1.02 | 0.75 | 1.38 |
| 7 | 1.11 | 1.00 | 1.22 | 1.07 | 0.92 | 1.25 | 1.20 | 0.86 | 1.68 |
| 8 | 1.03 | 0.93 | 1.14 | 1.02 | 0.87 | 1.21 | 0.91 | 0.65 | 1.27 |
| 9 | 1.13 | 1.00 | 1.28 | 1.10 | 0.90 | 1.34 | 1.11 | 0.82 | 1.52 |
| 10 | 1.05 | 0.95 | 1.17 | 1.06 | 0.91 | 1.23 | 0.86 | 0.59 | 1.25 |
| 11 | 1.04 | 0.94 | 1.14 | 1.03 | 0.88 | 1.20 | 1.11 | 0.82 | 1.51 |
| 12 | 1.02 | 0.93 | 1.13 | 1.12 | 0.96 | 1.31 | 0.77 | 0.58 | 1.03 |
| 13 | 1.04 | 0.93 | 1.16 | 1.09 | 0.92 | 1.30 | 0.94 | 0.64 | 1.38 |
| 14 | 1.12 | 1.00 | 1.26 | 1.12 | 0.95 | 1.33 | 1.09 | 0.73 | 1.62 |
| 15 | 1.03 | 0.93 | 1.13 | 1.01 | 0.87 | 1.18 | 1.45 | 0.96 | 2.19 |
| 16 | 1.06 | 0.95 | 1.20 | 1.11 | 0.93 | 1.32 | 0.94 | 0.68 | 1.30 |
| 17 | 1.19 | 1.05 | 1.35 | 1.17 | 0.97 | 1.42 | 1.02 | 0.63 | 1.63 |
| 18 | 0.95 | 0.85 | 1.05 | 0.98 | 0.83 | 1.16 | 1.13 | 0.84 | 1.52 |
| 19 | 1.13 | 1.01 | 1.26 | 1.15 | 0.96 | 1.37 | 1.30 | 0.94 | 1.79 |
| 20 | 1.06 | 0.96 | 1.17 | 1.06 | 0.91 | 1.23 | 0.95 | 0.64 | 1.39 |
| 21 | 1.15 | 1.03 | 1.28 | 1.15 | 0.98 | 1.36 | 1.05 | 0.71 | 1.54 |
| 22 | 1.04 | 0.93 | 1.15 | 1.01 | 0.85 | 1.21 | 1.08 | 0.76 | 1.53 |
| 23 | 1.26 | 1.14 | 1.40 | 1.23 | 1.05 | 1.46 | 1.50 | 1.08 | 2.08 |
| 24 | 1.06 | 0.95 | 1.19 | 1.08 | 0.90 | 1.29 | 1.06 | 0.72 | 1.56 |
| 25 | 1.13 | 1.01 | 1.26 | 1.11 | 0.93 | 1.31 | 1.48 | 0.95 | 2.31 |
| 26 | 1.05 | 0.94 | 1.18 | 1.06 | 0.89 | 1.26 | 0.91 | 0.66 | 1.24 |
| 27 | 0.96 | 0.87 | 1.07 | 0.95 | 0.81 | 1.10 | 0.97 | 0.70 | 1.34 |
| 28 | 1.08 | 0.98 | 1.19 | 1.06 | 0.92 | 1.23 | 1.39 | 0.92 | 2.09 |
| 29 | 0.91 | 0.81 | 1.02 | 0.91 | 0.76 | 1.10 | 1.03 | 0.72 | 1.47 |
| 30 | 1.13 | 1.01 | 1.27 | 1.18 | 0.99 | 1.41 | 0.93 | 0.63 | 1.38 |
| 31 | 1.03 | 0.92 | 1.14 | 1.00 | 0.85 | 1.18 | 1.11 | 0.77 | 1.61 |
| 32 | 1.10 | 1.00 | 1.22 | 1.15 | 0.99 | 1.35 | 1.03 | 0.74 | 1.43 |
| 33 | 1.08 | 0.98 | 1.20 | 1.08 | 0.92 | 1.27 | 1.11 | 0.77 | 1.62 |
| 34 | 1.00 | 0.90 | 1.12 | 0.96 | 0.82 | 1.14 | 1.33 | 0.88 | 2.00 |
| 35 | 1.02 | 0.92 | 1.13 | 0.99 | 0.85 | 1.17 | 1.17 | 0.85 | 1.60 |
| 36 | 1.10 | 0.99 | 1.21 | 1.13 | 0.97 | 1.32 | 0.90 | 0.66 | 1.25 |
| 37 | 1.11 | 1.00 | 1.24 | 1.17 | 0.98 | 1.39 | 1.03 | 0.75 | 1.43 |
| 38 | 0.99 | 0.89 | 1.10 | 0.92 | 0.78 | 1.09 | 1.13 | 0.82 | 1.57 |
| 39 | 1.04 | 0.94 | 1.14 | 1.10 | 0.93 | 1.28 | 1.00 | 0.74 | 1.35 |
| 40 | 0.94 | 0.85 | 1.04 | 0.91 | 0.77 | 1.07 | 1.08 | 0.73 | 1.58 |
| 41 | 1.16 | 1.03 | 1.30 | 1.12 | 0.94 | 1.34 | 1.31 | 0.85 | 2.02 |
| 42 | 1.32 | 1.17 | 1.48 | 1.30 | 1.09 | 1.56 | 1.94 | 1.22 | 3.09 |
| 43 | 1.04 | 0.95 | 1.15 | 1.05 | 0.90 | 1.22 | 0.86 | 0.63 | 1.17 |
| 44 | 1.04 | 0.94 | 1.16 | 1.11 | 0.94 | 1.31 | 0.86 | 0.61 | 1.22 |
| 45 | 0.97 | 0.88 | 1.07 | 0.94 | 0.80 | 1.10 | 0.89 | 0.65 | 1.22 |
| 46 | 0.97 | 0.87 | 1.07 | 0.94 | 0.80 | 1.10 | 1.01 | 0.73 | 1.40 |
| 47 | 1.11 | 1.00 | 1.23 | 1.10 | 0.94 | 1.29 | 1.01 | 0.73 | 1.40 |
| 48 | 1.14 | 1.03 | 1.25 | 1.22 | 1.04 | 1.43 | 0.97 | 0.73 | 1.31 |
| 49 | 1.18 | 1.06 | 1.30 | 1.12 | 0.95 | 1.31 | 1.38 | 0.98 | 1.96 |
| 50 | 1.04 | 0.93 | 1.16 | 1.00 | 0.85 | 1.19 | 1.26 | 0.91 | 1.75 |
| 51 | 1.00 | 0.90 | 1.10 | 1.00 | 0.86 | 1.16 | 1.11 | 0.83 | 1.50 |
| 52 | 1.06 | 0.96 | 1.16 | 1.09 | 0.94 | 1.25 | 1.02 | 0.70 | 1.48 |
| 53 | 1.03 | 0.94 | 1.13 | 1.03 | 0.89 | 1.19 | 1.02 | 0.80 | 1.30 |
| 54 | 1.01 | 0.91 | 1.11 | 1.04 | 0.88 | 1.23 | 0.95 | 0.70 | 1.28 |
| 55 | 1.10 | 1.00 | 1.22 | 1.09 | 0.93 | 1.27 | 1.15 | 0.87 | 1.51 |
| 56 | 1.08 | 0.98 | 1.20 | 1.17 | 1.00 | 1.37 | 0.92 | 0.68 | 1.24 |
| 57 | 1.06 | 0.97 | 1.17 | 1.10 | 0.95 | 1.27 | 0.88 | 0.66 | 1.19 |
| 58 | 1.02 | 0.93 | 1.12 | 1.00 | 0.86 | 1.16 | 1.07 | 0.77 | 1.50 |
| 59 | 0.99 | 0.89 | 1.11 | 1.00 | 0.84 | 1.19 | 1.07 | 0.79 | 1.45 |
| 60 | 0.95 | 0.86 | 1.05 | 0.91 | 0.78 | 1.06 | 1.23 | 0.89 | 1.70 |
| 61 | 1.24 | 1.12 | 1.37 | 1.30 | 1.10 | 1.53 | 1.14 | 0.87 | 1.50 |
| 62 | 0.93 | 0.84 | 1.04 | 0.94 | 0.79 | 1.11 | 1.01 | 0.75 | 1.36 |
| 63 | 1.03 | 0.93 | 1.13 | 0.98 | 0.84 | 1.14 | 1.29 | 0.96 | 1.73 |
| 64 | 1.14 | 1.03 | 1.26 | 1.16 | 0.99 | 1.35 | 0.95 | 0.69 | 1.30 |
| 65 | 1.04 | 0.95 | 1.15 | 1.04 | 0.89 | 1.21 | 1.14 | 0.82 | 1.58 |
| 66 | 0.99 | 0.90 | 1.09 | 1.04 | 0.89 | 1.22 | 0.79 | 0.59 | 1.06 |
| 67 | 1.20 | 1.10 | 1.32 | 1.16 | 1.00 | 1.34 | 1.17 | 0.87 | 1.56 |
| 68 | 1.14 | 1.04 | 1.26 | 1.12 | 0.96 | 1.30 | 1.19 | 0.85 | 1.66 |
| 69 | 1.07 | 0.97 | 1.18 | 1.11 | 0.96 | 1.29 | 0.81 | 0.58 | 1.12 |
| 70 | 0.89 | 0.80 | 0.98 | 0.87 | 0.75 | 1.02 | 1.25 | 0.89 | 1.76 |
| 71 | 1.08 | 0.98 | 1.18 | 1.08 | 0.93 | 1.26 | 0.94 | 0.72 | 1.24 |
| 72 | 1.04 | 0.94 | 1.15 | 1.05 | 0.90 | 1.23 | 0.95 | 0.69 | 1.30 |
| 73 | 0.99 | 0.90 | 1.10 | 0.98 | 0.83 | 1.15 | 1.11 | 0.82 | 1.50 |
| 74 | 1.01 | 0.91 | 1.11 | 0.99 | 0.84 | 1.15 | 0.98 | 0.71 | 1.34 |
| 75 | 1.05 | 0.96 | 1.16 | 1.05 | 0.90 | 1.22 | 1.00 | 0.73 | 1.37 |
| 76 | 1.09 | 0.99 | 1.19 | 1.11 | 0.96 | 1.29 | 1.10 | 0.84 | 1.44 |
| 77 | 1.04 | 0.94 | 1.14 | 1.05 | 0.90 | 1.21 | 0.97 | 0.75 | 1.26 |
| 78 | 1.01 | 0.92 | 1.12 | 1.02 | 0.87 | 1.18 | 1.11 | 0.82 | 1.50 |
| 79 | 1.15 | 1.03 | 1.28 | 1.14 | 0.96 | 1.36 | 1.24 | 0.91 | 1.70 |
| 80 | 1.07 | 0.98 | 1.17 | 1.11 | 0.96 | 1.28 | 0.93 | 0.71 | 1.22 |
| 81 | 1.07 | 0.98 | 1.18 | 1.05 | 0.91 | 1.21 | 1.24 | 0.91 | 1.70 |
| 82 | 1.07 | 0.98 | 1.18 | 1.05 | 0.91 | 1.21 | 1.15 | 0.82 | 1.61 |
| 83 | 1.13 | 1.03 | 1.24 | 1.06 | 0.91 | 1.24 | 1.40 | 1.08 | 1.83 |
| 84 | 1.07 | 0.98 | 1.16 | 1.06 | 0.92 | 1.21 | 1.02 | 0.79 | 1.30 |
| 85 | 1.11 | 1.01 | 1.21 | 1.20 | 1.04 | 1.39 | 0.88 | 0.68 | 1.13 |
| 86 | 1.04 | 0.95 | 1.15 | 1.05 | 0.90 | 1.22 | 1.00 | 0.74 | 1.35 |
| 87 | 1.00 | 0.91 | 1.10 | 0.96 | 0.83 | 1.13 | 1.03 | 0.80 | 1.33 |
| 88 | 1.05 | 0.96 | 1.15 | 1.03 | 0.89 | 1.20 | 1.05 | 0.80 | 1.38 |
| 89 | 1.02 | 0.94 | 1.12 | 1.04 | 0.91 | 1.19 | 1.02 | 0.77 | 1.36 |
| 90 | 1.02 | 0.93 | 1.11 | 0.98 | 0.86 | 1.13 | 0.95 | 0.68 | 1.32 |
| 91 | 1.06 | 0.97 | 1.15 | 1.10 | 0.96 | 1.26 | 0.92 | 0.71 | 1.19 |
| 92 | 1.24 | 1.13 | 1.36 | 1.23 | 1.06 | 1.43 | 1.23 | 0.93 | 1.64 |
| 93 | 1.17 | 1.06 | 1.28 | 1.19 | 1.03 | 1.39 | 1.04 | 0.79 | 1.37 |
| 94 | 1.06 | 0.97 | 1.15 | 1.08 | 0.94 | 1.23 | 0.98 | 0.75 | 1.30 |
| 95 | 1.02 | 0.94 | 1.10 | 1.03 | 0.91 | 1.18 | 0.97 | 0.75 | 1.25 |
| 96 | 1.00 | 0.92 | 1.08 | 0.94 | 0.83 | 1.08 | 1.33 | 1.03 | 1.71 |
| 97 | 0.99 | 0.91 | 1.08 | 0.92 | 0.80 | 1.06 | 1.29 | 1.02 | 1.64 |
| 98 | 1.14 | 1.05 | 1.24 | 1.10 | 0.96 | 1.26 | 1.25 | 0.99 | 1.58 |
| 99 | 1.10 | 1.02 | 1.20 | 1.06 | 0.93 | 1.21 | 1.21 | 0.96 | 1.51 |
| 100 | 0.99 | 0.92 | 1.07 | 0.99 | 0.88 | 1.12 | 1.03 | 0.85 | 1.24 |

**Table S9.** Results of linear Mendelian randomization (MR) sensitivity analyses investigating associations of genetically proxied blood pressure (instruments selected from non-UK Biobank studies without adjustment for body mass index) with risk of cardiovascular disease outcomes. IVW: random effects inverse-variance weighted model.

| **Exposure** | **Method** | **Cardiovascular disease** | | | **Coronary artery disease** | | | **Stroke** | | |
| --- | --- | --- | --- | --- | --- | --- | --- | --- | --- | --- |
|  |  | **Hazard ratio** | **95% confidence interval** | **P** | **Hazard ratio** | **95% confidence interval** | **P** | **Hazard ratio** | **95% confidence interval** | **P** |
| Systolic blood pressure (per 10mmHg increase) | Allele score | 1.47 | 1.20-1.80 | 1.45E-3 | 1.48 | 1.19-1.85 | 2.51E-4 | 1.52 | 1.28-1.81 | 1.44E-4 |
|  | IVW | 1.57 | 1.22-2.01 | 4.45E-4 | 1.59 | 1.19-2.08 | 1.40E-4 | 1.56 | 1.27-1.94 | 3.15E-5 |
|  | Weighted median | 1.59 | 1.24-1.99 | 2.73E-4 | 1.60 | 1.17-2.04 | 1.54E-5 | 1.58 | 1.28-1.95 | 2.15E-3 |
|  | MR-PRESSO | 1.64 | 1.38-1.95 | 2.29E-4 | 1.56 | 1.27-1.93 | 1.74E-3 | 1.57 | 1.28-1.93 | 5.95E-4 |
|  | MR-Egger intercept |  | | 0.34 |  | | 0.28 |  | | 0.39 |
|  | MR-Egger | 0.95 | 0.38-2.40 | 0.92 | 0.99 | 0.89-1.09 | 0.38 | 1.94 | 0.76-4.92 | 0.19 |
| Diastolic blood pressure (per 5mmHg increase) | Allele score | 1.43 | 1.27-1.62 | 4.01E-6 | 1.44 | 1.27-1.64 | 8.03E-6 | 1.32 | 1.20-1.46 | 1.11E-5 |
|  | IVW | 1.62 | 1.37-1.92 | 2.70E-8 | 1.64 | 1.37-1.98 | 1.14E-7 | 1.32 | 1.15-1.52 | 1.22E-4 |
|  | Weighted median | 1.32 | 1.19-1.48 | 3.74E-7 | 1.35 | 1.19-1.52 | 2.58E-6 | 1.40 | 1.16-1.69 | 5.54E-4 |
|  | MR-PRESSO | 1.43 | 1.24-1.63 | 1.20E-4 | 1.48 | 1.26-1.74 | 3.50E-4 | 1.32 | 1.15-1.51 | 6.94E-4 |
|  | MR-Egger intercept |  | | 0.89 |  | | 0.98 |  | | 0.94 |
|  | MR-Egger | 1.49 | 0.82-2.71 | 0.20 | 1.58 | 0.82-3.03 | 0.18 | 1.35 | 0.85-2.15 | 0.20 |

**Figure S1.** Scatter plot of association estimates for genetically proxied systolic blood pressure (SBP) and (A) incident cardiovascular disease (CVD) risk, (B) incident coronary artery disease (CAD) risk, and (C) incident stroke risk. Mendelian randomization (MR) estimates from the inverse-variance weighted, weighted median, weighted mode, simple mode and MR Egger methods are provided. For each single-nucleotide polymorphism (SNP; N=253), the effect and the standard error on the exposure (SBP) and the outcome are plotted.


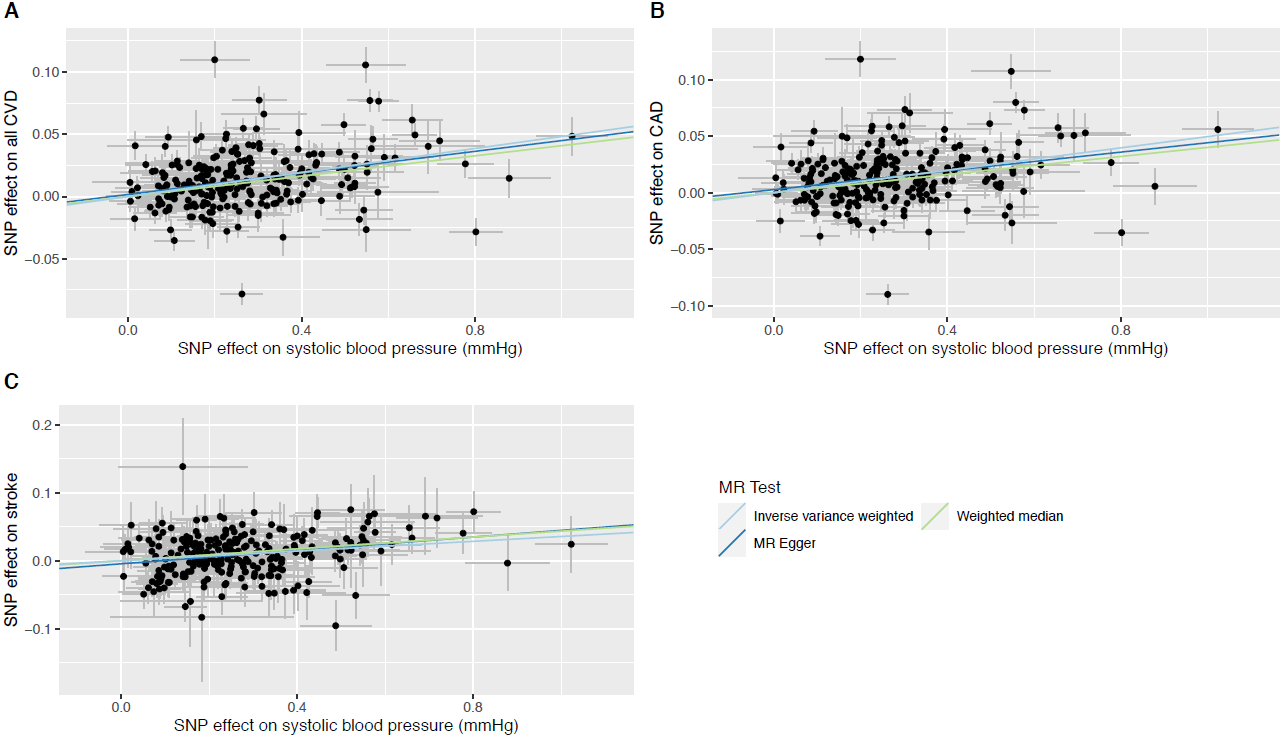


**Figure S2.** Scatter plot of association estimates for genetically proxied diastolic blood pressure (DBP) and (A) incident cardiovascular disease (CVD) risk, (B) incident coronary artery disease (CAD) risk, and (C) incident stroke risk. Mendelian randomization (MR) estimates from the inverse-variance weighted, weighted median, weighted mode, simple mode and MR-Egger methods are provided. For each single-nucleotide polymorphism (SNP; N=253), the effect and the standard error on the exposure (DBP) and the outcome are plotted.
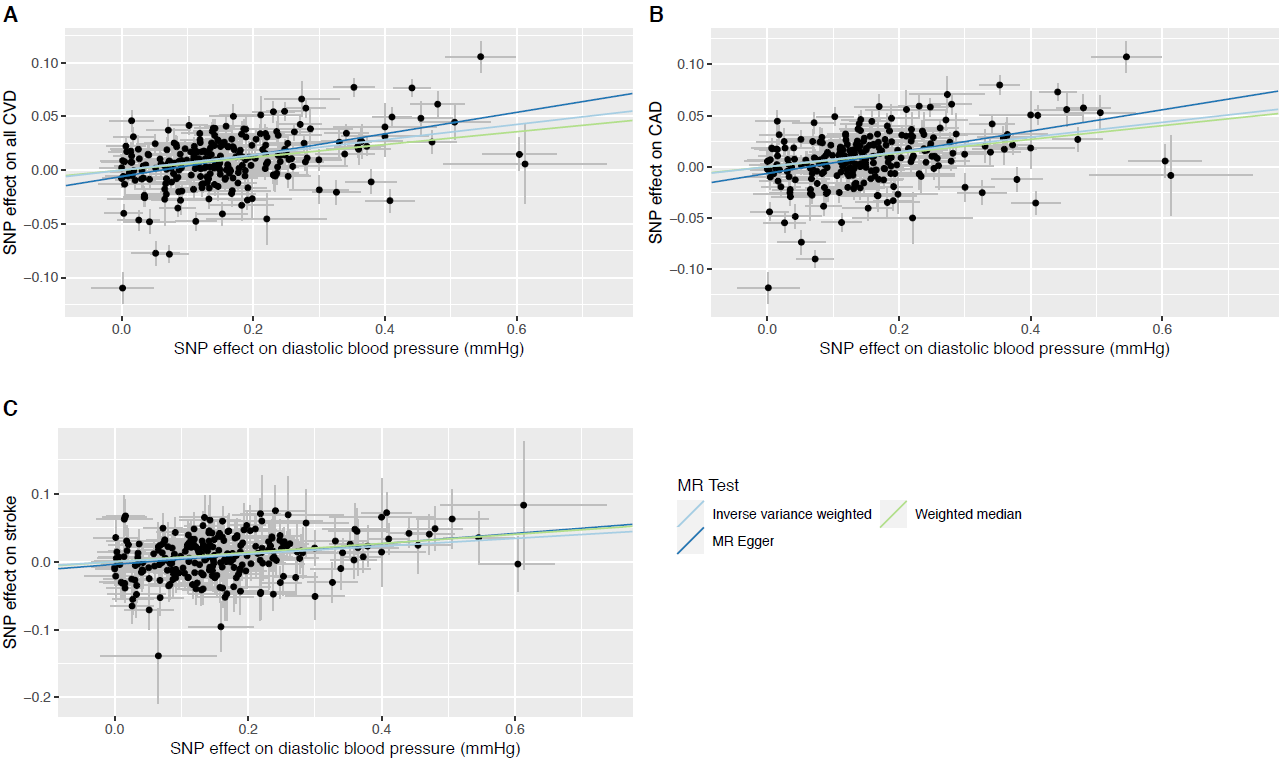


**Figure S3.** Non-linear Mendelian randomization analysis considering genetically proxied systolic blood pressure (SBP) and incident cardiovascular outcomes using inverse-probability weighting: (A) all incident cardiovascular disease (CVD), (B) incident coronary artery disease (CAD) and (C) incident stroke. Displayed on the x-axis are SBP values in mmHg. The y-axis shows the hazard ratio for the respective incident cardiovascular event. Reference is set to a population mean SBP value of 136.5mmHg. Grey lines depict the 95% confidence interval.

**
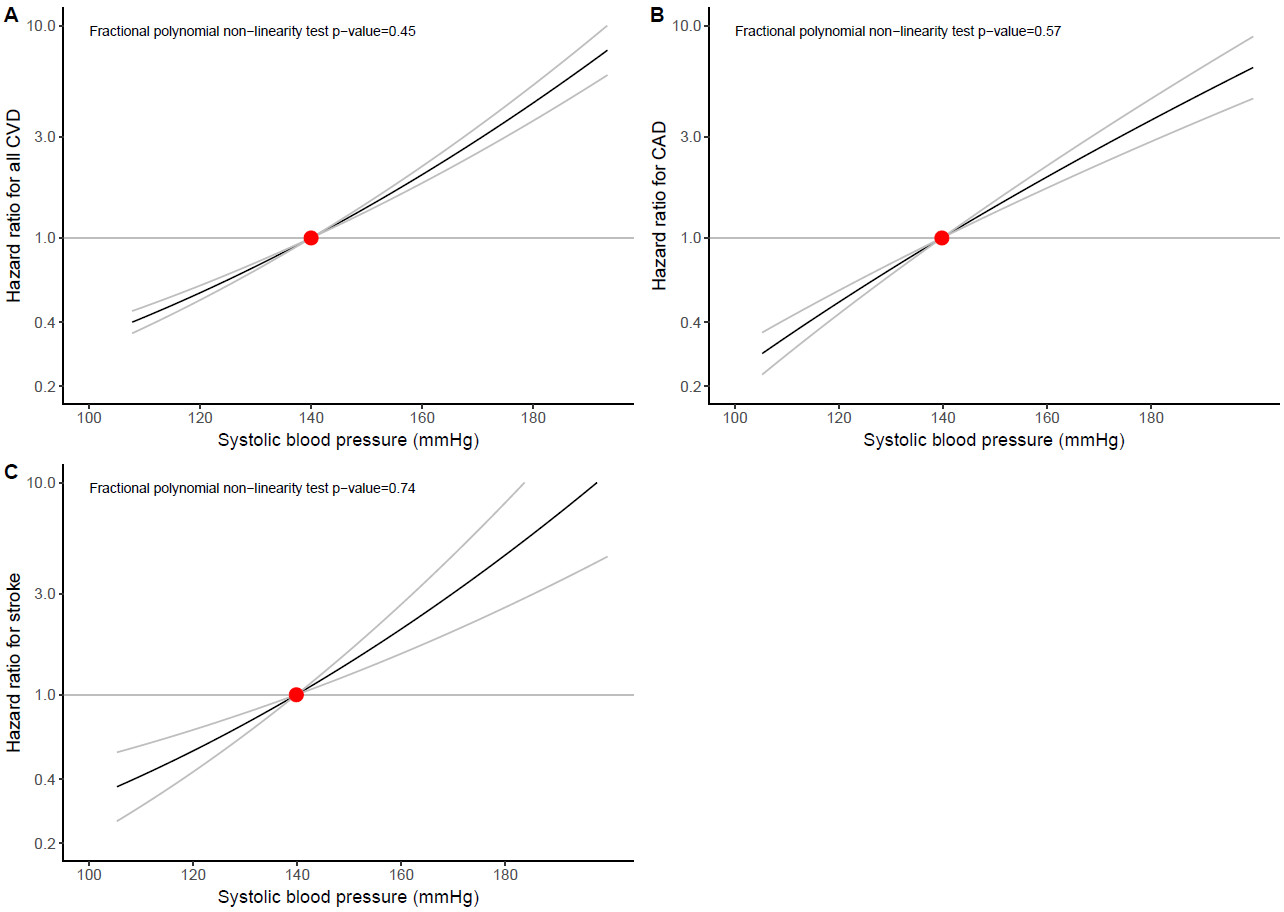
**

**Figure S4.** Non-linear Mendelian randomization analysis considering genetically proxied diastolic blood pressure (DBP) and incident cardiovascular outcomes using inverse-probability weighting: (A) all incident cardiovascular disease (CVD), (B) incident coronary artery disease (CAD) and (C) incident stroke. Displayed on the x-axis are DBP values in mmHg. The y-axis shows the hazard ratio for the respective incident cardiovascular event. Reference is set to a population mean DBP value of 81.8mmHg. Grey lines depict the 95% confidence interval.

**
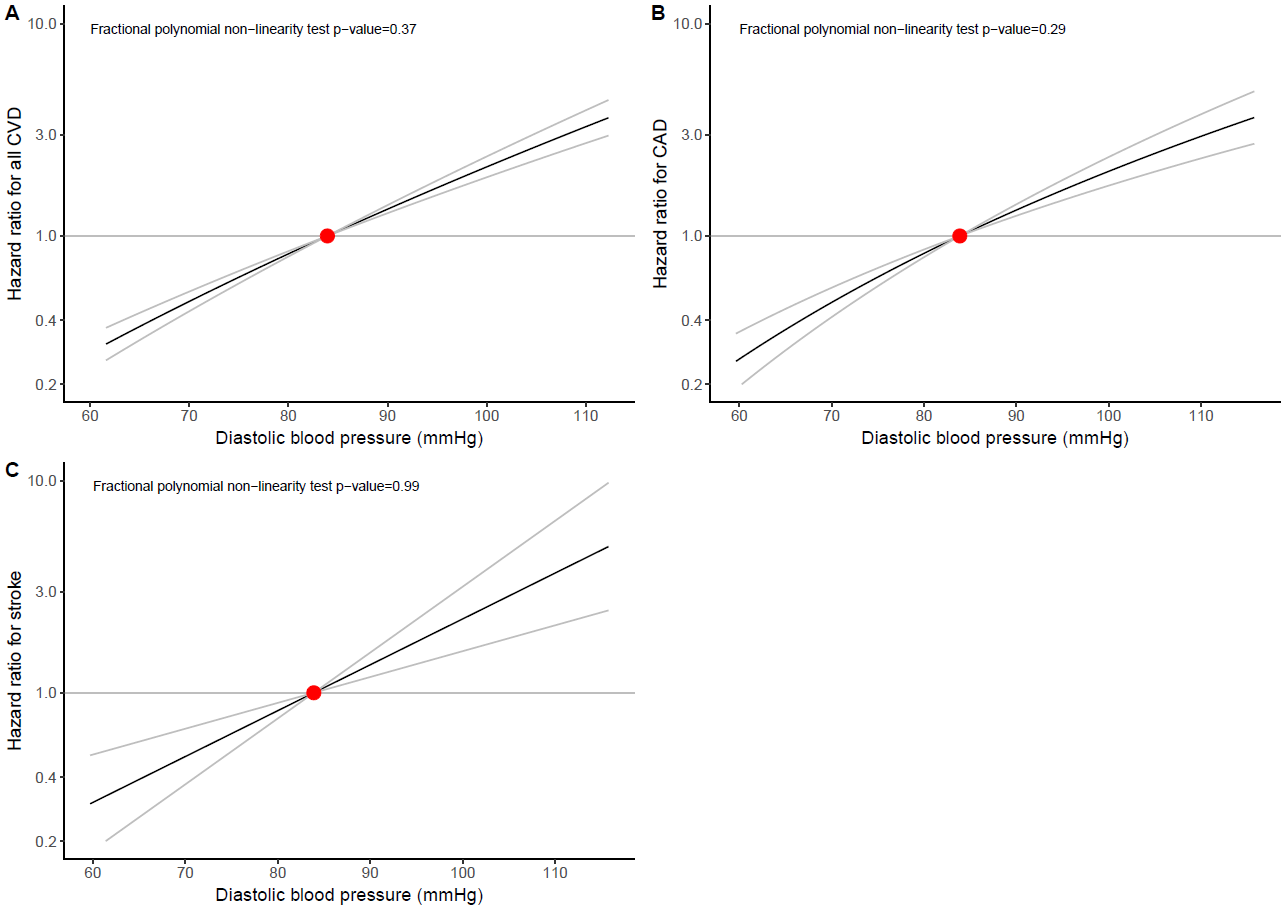
**

**
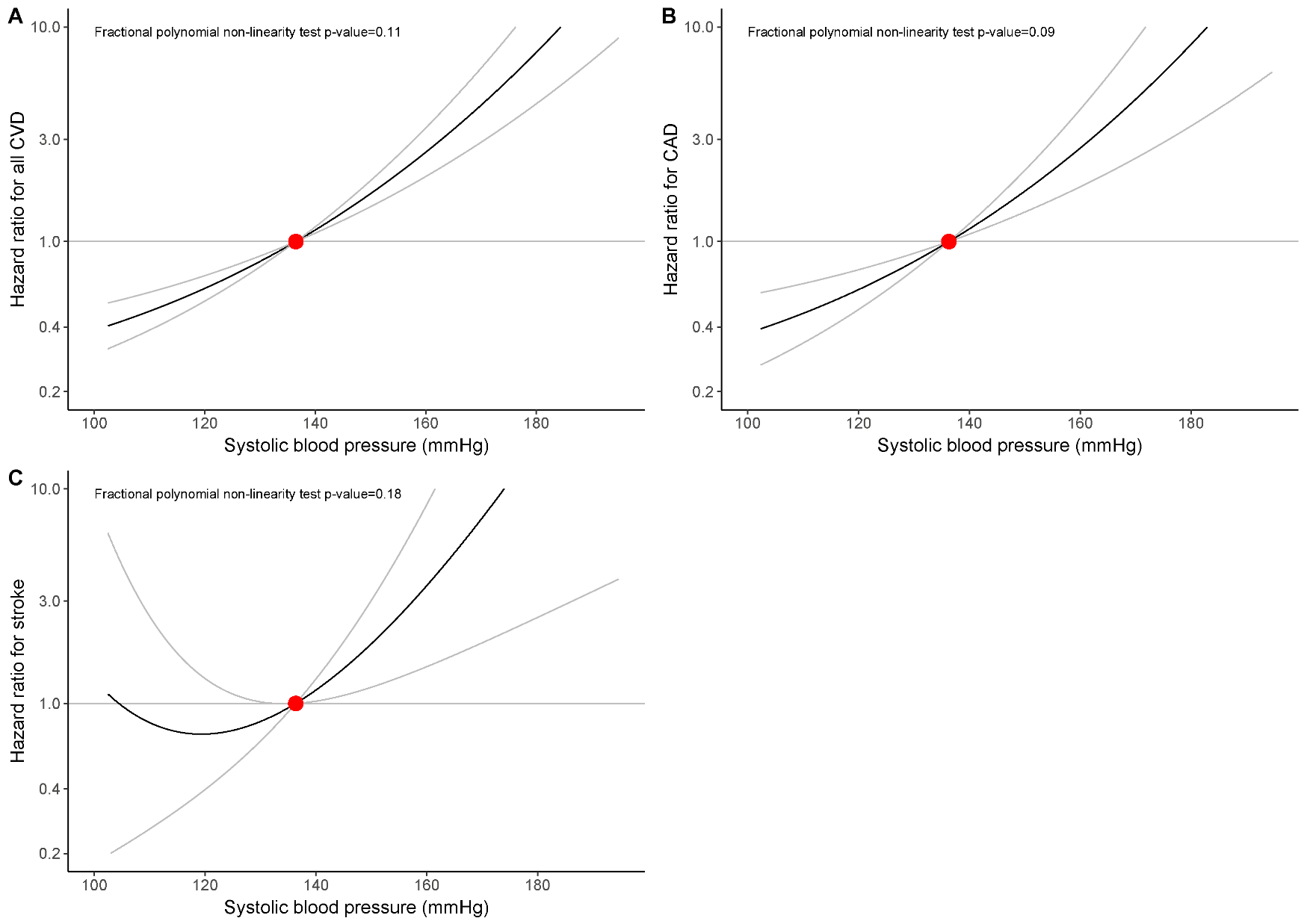
****Figure S5.** Non-linear Mendelian randomization analysis considering genetically proxied systolic blood pressure (SBP) and incident cardiovascular outcomes using an instrument set derived from non-UK Biobank studies without adjustment for body mass index: (A) all incident cardiovascular disease (CVD), (B) incident coronary artery disease (CAD) and (C) incident stroke. Displayed on the x-axis are SBP values in mmHg. The y-axis shows the hazard ratio for the respective incident cardiovascular event. Reference is set to a population mean SBP value of 136.5mmHg. Grey lines depict the 95% confidence interval.

**
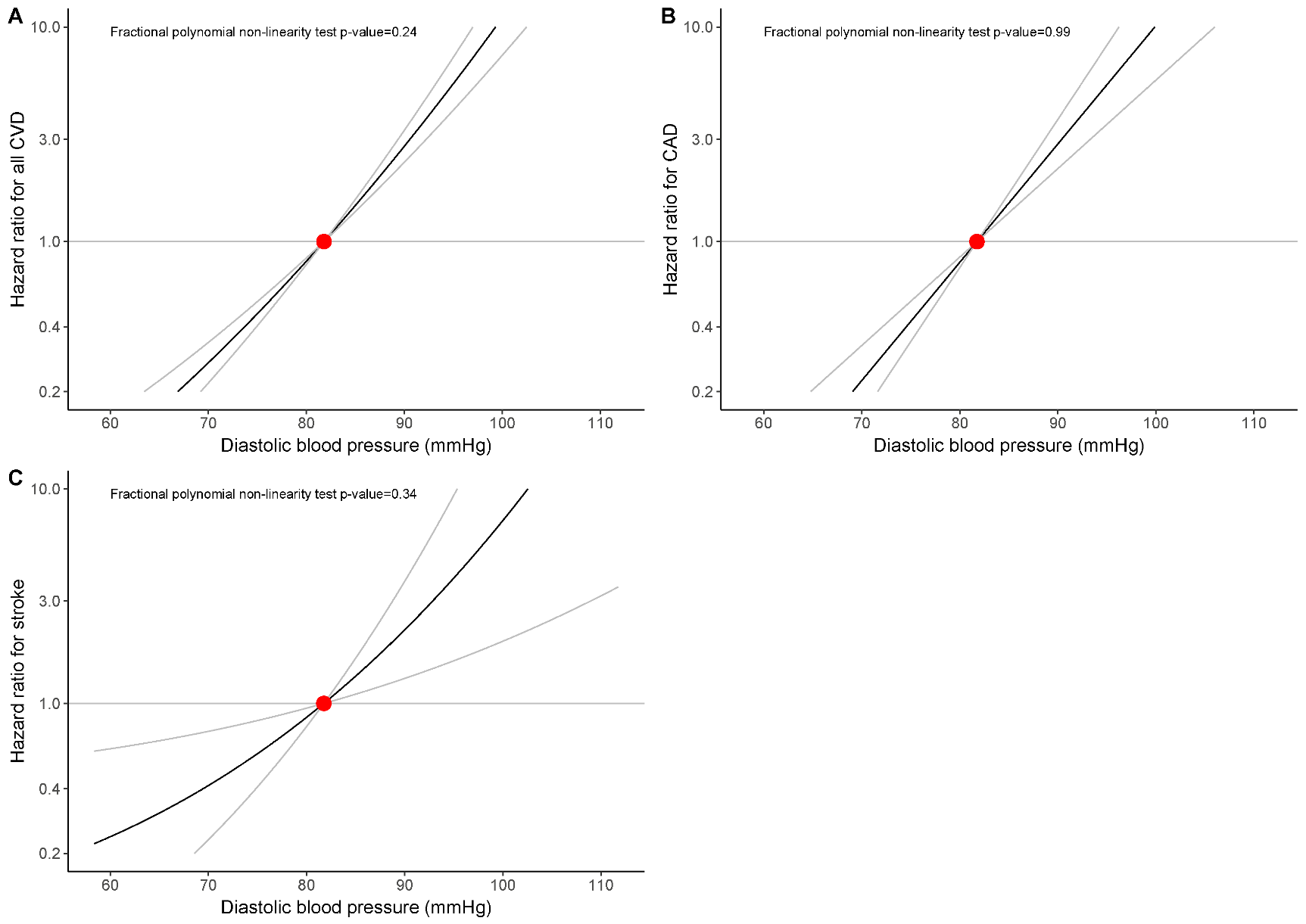
****Figure S6.** Non-linear Mendelian randomization analysis considering genetically proxied diastolic blood pressure (DBP) and incident cardiovascular outcomes using an instrument set derived from non-UK Biobank studies without adjustment for body mass index: (A) all incident cardiovascular disease (CVD), (B) incident coronary artery disease (CAD) and (C) incident stroke. Displayed on the x-axis are DBP values in mmHg. The y-axis shows the hazard ratio for the respective incident cardiovascular event. Reference is set to a population mean SBP value of 81.8mmHg. Grey lines depict the 95% confidence interval.
